# EIF4B Ser93 phosphorylation by ERK2 promotes epithelial-mesenchymal transition to drive colorectal cancer metastasis

**DOI:** 10.1038/s41419-025-08375-5

**Published:** 2026-01-05

**Authors:** Siqi Wen, Min Lin, Man Zhang, Zhao Li, Jinchi Chen, Bei Yi, Dejun Liu, Ruiqi Chen, Tianyu Chen, Rong Liang, Wei Jiang

**Affiliations:** 1 Department of Experimental Research, Guangxi Medical University Cancer Hospital, Nanning, China; 2 Department of Radiation Oncology, Guangxi Medical University Cancer Hospital, Nanning, China; 3 Department of Gastrointestinal Surgery, Guangxi Clinical Research Center for Colorectal Cancer, Guangxi Medical University Cancer Hospital, Nanning, China

**Keywords:** Colorectal cancer, Phosphorylation

## Abstract

Colorectal cancer (CRC) is the third most common malignant tumor and the second leading cause of cancer-related mortality globally. Epithelial to mesenchymal transition (EMT) contributes to CRC metastasis and poor prognosis. Aberrant protein phosphorylation is implicated in CRC progression, warranting further investigation into its molecular mechanisms. Herein, we have identified significant alterations in protein phosphorylation associated with CRC through tandem mass tag (TMT) label-based phosphoproteomic analysis. The functions and enriched signaling pathways of these proteins were predominantly linked to the EMT process. Notably, the phosphorylation of eIF4B at Ser93 exhibited the most pronounced increase in CRC, a finding that was further validated in CRC tissues and cell lines by a newly generated antibody targeting eIF4B Ser93 phosphorylation. Phosphorylation of eIF4B Ser93 promoted CRC progression and metastasis both in vitro and in vivo. Mechanistically, eIF4B Ser93 phosphorylation decreased ubiquitination-mediated eIF4B degradation and enhanced its translation activity, through which it facilitated the translation of mesenchymal markers. Additionally, ERK2 directly phosphorylated eIF4B at Ser93, while inhibiting this phosphorylation is essential for the anti-cancer efficacy of the ERK2 inhibitor, Vx-11e. Together, the phosphorylation of eIF4B Ser93 driven by ERK2 promotes CRC growth and metastasis through the activation of EMT. Our findings indicate a novel therapeutic target and provide promising strategies for clinical intervention in human CRC.

## Introduction

Colorectal cancer (CRC) is the most prevalent malignant tumor of the gastrointestinal tract, with the second leading cause of cancer-related mortality, currently ranking as the third most common type of cancer globally [1]. As the quality of life improves, the incidence of CRC is evidently increasing worldwide [2, 3]. Notably, due to its large population, China reports the highest number of new CRC cases and CRC-related deaths globally [4, 5]. Metastases are the primary cause of mortality among patients diagnosed with CRC [6, 7]. In advanced stages of the disease, the 5-year survival rate for patients with metastases declines to less than 15%, and the median survival duration is under 30 months [8]. Consequently, more in depth investigations into the metastatic mechanisms of CRC is of great significance.

Epithelial-mesenchymal transition (EMT) is a crucial process in the metastases of CRC, which is closely associated with patient poor prognosis [9]. However, the underlying mechanisms of EMT remain little understood. EMT is characterized by the loss of epithelial cell properties and the gain of mesenchymal cell properties, which leads to increasing cellular motility and the acquisition of invasiveness [10, 11]. This transition typically serves as the initial step in the invasion-metastasis cascade of CRC cells [12]. Studies showed that the EMT status of CRC promotes the formation of cell clusters during dissemination, thereby enhancing their metastatic potential, and colonization capabilities at distant sites [13]. Nevertheless, due to the inherent plasticity and heterogeneity of EMT pathway, investigations into the intricate mechanisms that govern EMT in CRC are still not fully elucidated [14].

Dysregulated mRNA translation generally contributes to the increased synthesis of oncoproteins, thereby facilitating tumor cells proliferation and metastasis [15]. In eukaryotes, translation initiation is the most intricate of all phases, requiring meticulous regulation by a variety of initiation factors [16]. Eukaryotic initiation factor 4B (eIF4B), which acts as an RNA chaperone, is integral to the recruitment of ribosomes and the scanning during translation initiation [17]. The phosphorylation of eIF4B has been shown to enhance translation activity, which is critical for the regulation of translation initiation in eukaryotes [18, 19]. The phosphorylation of eIF4B has been identified to play pro-oncogenic roles in multiple cancers [20]. eIF4B Ser422 phosphorylation is associated with increased matrix metalloproteinases (MMPs) in the extracellular matrix in CRC and the upregulation of EMT transcription factors ZEB1/ZEB2 in lymphangioleiomyomatosis (LAM) [21, 22]. Thus, it is clear that eIF4B phosphorylation is pivotal in the advancement of cancers by affecting the translation of pro-oncogenic factors. However, the specific regulatory role of eIF4B phosphorylation in the metastasis of CRC remains to be fully elucidated.

The MAPK/ERK signaling pathway is constantly activated in CRC, driven by genetic mutations and the tumor microenvironment, such as gut microbiota and immune cells, which in turn facilitates tumor progression [23–25]. Inhibition of this pathway has been shown to reduce liver metastasis of CRC and enhance the sensitivity of CRC cells to oxaliplatin treatment [26]. Notably, researchers reported that ribosomal S6 kinase (RSK) downstream of the MAPK/ERK signaling was able to phosphorylate eIF4B at Ser422, which enhanced the helicase activity of eIF4A and subsequently improved the translation efficiency of eIF4B on target mRNA [19, 27]. These evidences support that ERK pathway involved in the regulation of both CRC progression and eIF4B phosphorylation [28].

Here, we discovered that eIF4B Ser93 phosphorylation promotes the metastasis of CRC by enhancing the stability of eIF4B and increasing the translation of EMT mesenchymal markers. ERK2 participant in CRC EMT and progression through directly phosphorylating eIF4B Ser93 phosphorylation. Our findings provide a novel molecular mechanism underlying the occurrence of EMT in CRC and contribute to a deeper understanding of the role of eIF4B phosphorylation in CRC progression.

## Results

### The protein phosphorylation level in colorectal cancer tisssues by phosphoproteomic analysis

**Fig. 1:**
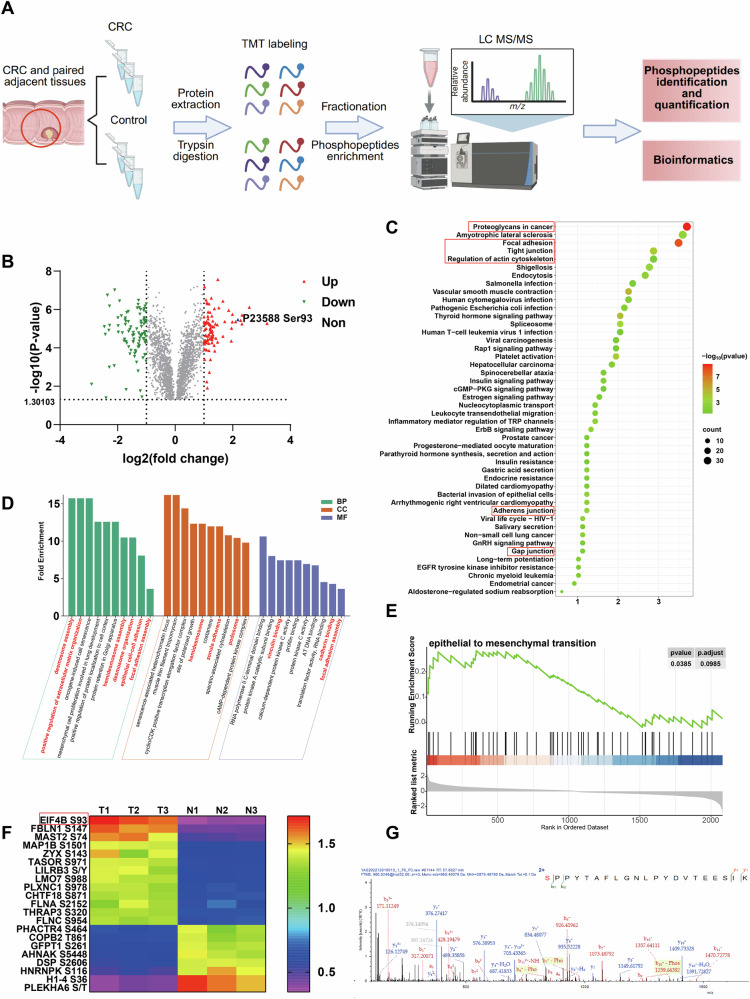
The protein phosphorylation level in colorectal cancer tisssues by phosphoproteomic analysis. **A** Schematic illustration of TMT-labeled quantitative phosphoproteomic used for the detection of differentially expressed phosphoproteins (DPPs) in CRC tissues compared to the normal tissues. **B** Volcano plot showed the phosphorylated alteration of DPPs in CRC tissues compared to the controls. The blue up triangle represents the phosphorylated alteration of eIF4B (Uniprot ID: P23588) at Ser93. **C**, **D** KEGG and GO analysis was conducted to investigate the signaling pathways and cell functions which phosphorylated alteration DPPs enriched on. **E** GSEA analysis showed the significant enrichment of EMT in proteins phosphorylation increasing group. **F** Heat map of all DPPs meeting∣log2(T/N)∣ > 1, ion Score>50 and site probability above 90. Data were presented as normalized expression values of 3 independent replicates. The color key represents the normalized values: blue (low) to red (high). **G** Mass spectrometry analysis of eIF4B tryptic peptides (Ser93-Lys113).

To identify the potential aberrant phosphorylation involved CRC progression, we performed phosphoproteomic analysis and obtained DPPs from CRC and the control tissues (Fig. 1A). A total of 2099 phosphosites within 1280 DPPs were identified in CRC tissues compared to controls (*p* < 0.05). Among these, the phosphorylation levels of 87 phosphosites within 79 DPPs were significantly increased (log2(T/N) > 1), while 101 phosphosites within 88 DPPs were significantly decreased (log2(T/N) <-1, Fig. 1B). KEGG pathway and gene ontology (GO) analysis were used to define the potential function of these DPPs phosphorylation alteration in CRC. Many of these DPPs were enriched on signaling pathways associated with cell migration and adhesion, such as proteoglycans in cancer, focal adhesion, tight junction, regulate of actin cytoskeleton, adherens junction, and gap junction (Fig. 1C). Several molecular functions related to cell adhesion, such as vinculin binding, cadherin binding, and focal adhesion assembly were also involved with many DPPs (Fig. 1D). Gene Set Enrichment Analysis (GSEA) of DPPs showed that proteins phosphorylation increasing group were significantly enriched on EMT (Fig. 1E) and other cell adhesion and motility related functions (Fig. S1). To explore the key DPPs involved in CRC development, we selected 21 DPPs that meeting∣log2(T/N)∣ > 1 (significant difference), ion Score>50 (high quality modified peptide profiles) and site probability above 90 (high credibility of the phosphosites) from all the DPPs. As shown in the heat map, eIF4B Ser93 is the top increase phosphorylation among the key DPPs (Fig. 1F). Furthermore, the MS/MS spectrum derived from the phosphorated eIF4B Ser93 showed high-quality with excellent signal-to-noise ratio, and extensive product ions b-ion and y-ion series (b1, b2, b3, b4, y1, y2, y3, and y4) (Fig. 1G). In summary, the abnormal proteins phosphorylation in CRC tissues are mainly involved in cell migration, adhesion, and EMT. Notably, the phosphorylation of eIF4B Ser93 significantly increases among the key DPPs identified through phosphoproteomic analysis.

### The levels of eIF4B and its Ser93 phosphorylation increase in CRC tissues and cell lines

**Fig. 2:**
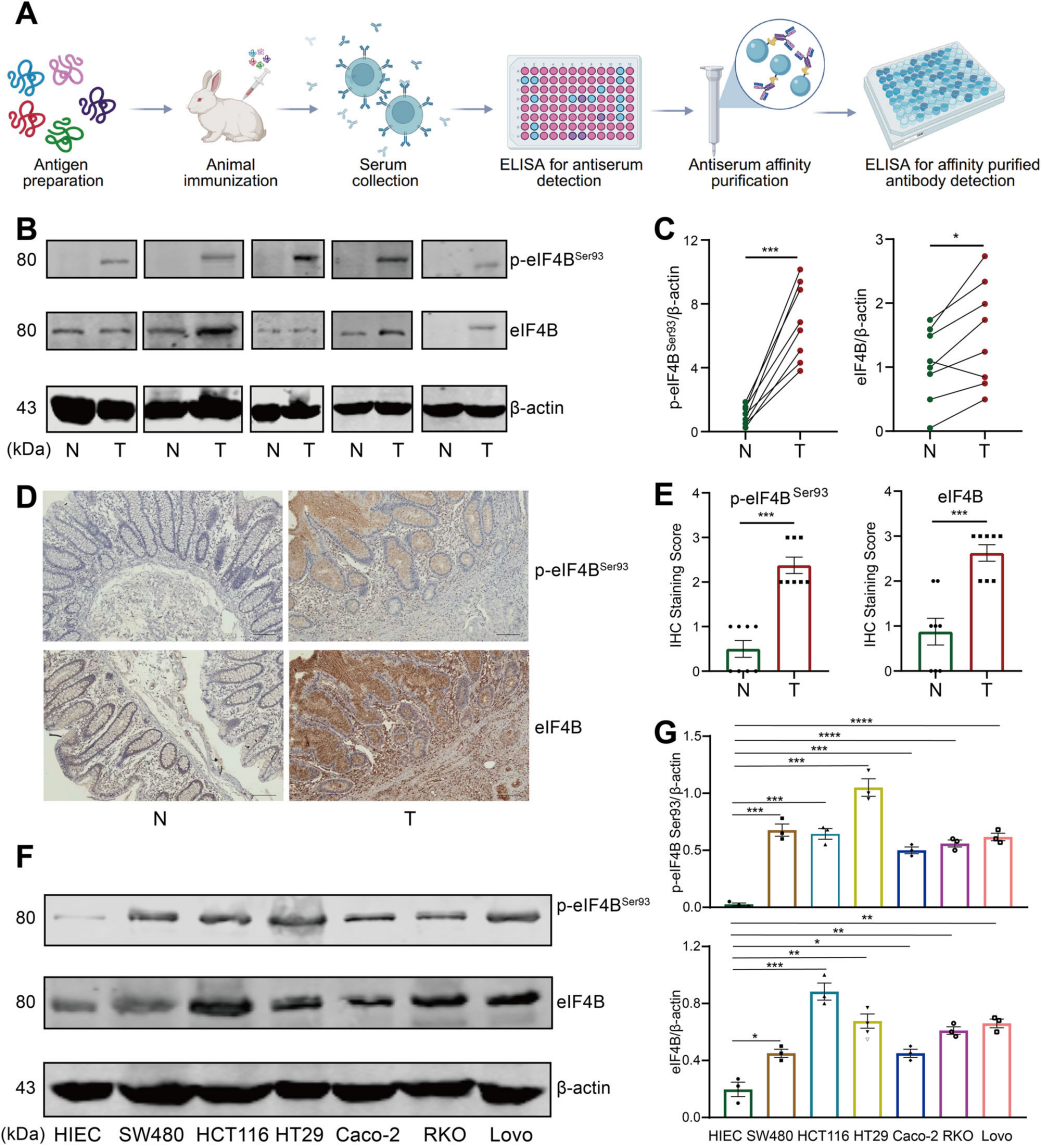
The levels of eIF4B and its Ser93 phosphorylation increase in CRC tissues and cell lines. **A** The manufacturing flowchart for the specific antibody targeting eIF4B Ser93 phosphorylation. **B**, **C** Western blotting analysis of eIF4B and p-eIF4B^Ser93^ in CRC tissues with the paired normal tissues as control (*n* = 8). The quantitative results of eIF4B expression and Ser93 phosphorylation levels were evaluated by Image J, and data were analyzed by two tailed paired Student’s t-test and presented as normalized expression values. **D** Immunohistochemistry of eIF4B and p-eIF4B^Ser93^ in tissue slices generated from human CRC primary tumor samples with the paired normal tissues as control. **E** The score of the staining intensity of eIF4B and p-eIF4B^Ser93^ in tissue slices of CRC and the controls. **F**, **G** The expression levels of eIF4B and its Ser93 phosphorylation in CRC cell lines were determined by western blotting. The quantitative results were evaluated by Image J, and data were presented as normalized values. Error bars represent mean ± SEM from at least three independent experiments. *P* values were determined by two-sided unpaired Student t test. *P < 0.05, **P < 0.01, ***P < 0.001, and ****P < 0.0001.

Given that proteomic analysis indicated a significant elevation in the phosphorylation level of eIF4B at Ser93, we therefore focused our investigation on eIF4B, specifically its phosphorylation at this site. The phosphor-specific antibody of eIF4B phospho-Ser93 was generated to further detect eIF4B Ser93 phosphorylation (Fig. 2A), which showed non-reaction with eIF4B non-phosphorylated peptides SRLPKSPPYTAC (88–98 aa) (Table S3) but sensitive to the SRLPK(p-)SPPYTAC (Table S4). To verify the result of phosphoproteomic analysis, we further compared the phosphorylation level of eIF4B Ser93 between CRC tissues and the paired normal tissues by western blotting and immunohistochemistry using this specific antibody. EIF4B Ser93 phosphorylation significantly increased in CRC, in line with the result of phosphoproteomic analysis (Fig. 2B–E). Similarly, the protein level of eIF4B also increased in CRC tissues (Fig. 2B–E). Furthermore, we also found that the phosphorylation of eIF4B Ser93 as well as the protein level of eIF4B increased in human CRC cell lines compared with the normal human intestinal epithelial cells (HIEC) in vitro (Fig. 2F, G). Conclusively, we developed a phosphor-specific antibody of eIF4B Ser93 to further verify the increase level of eIF4B Ser93 phosphorylation in both CRC tissues and cell lines.

### eIF4B knockdown decreased the proliferation, migration, and invasion of CRC cells, while eIF4B Ser93 phosphorylation enhanced these abilities in vitro

**Fig. 3:**
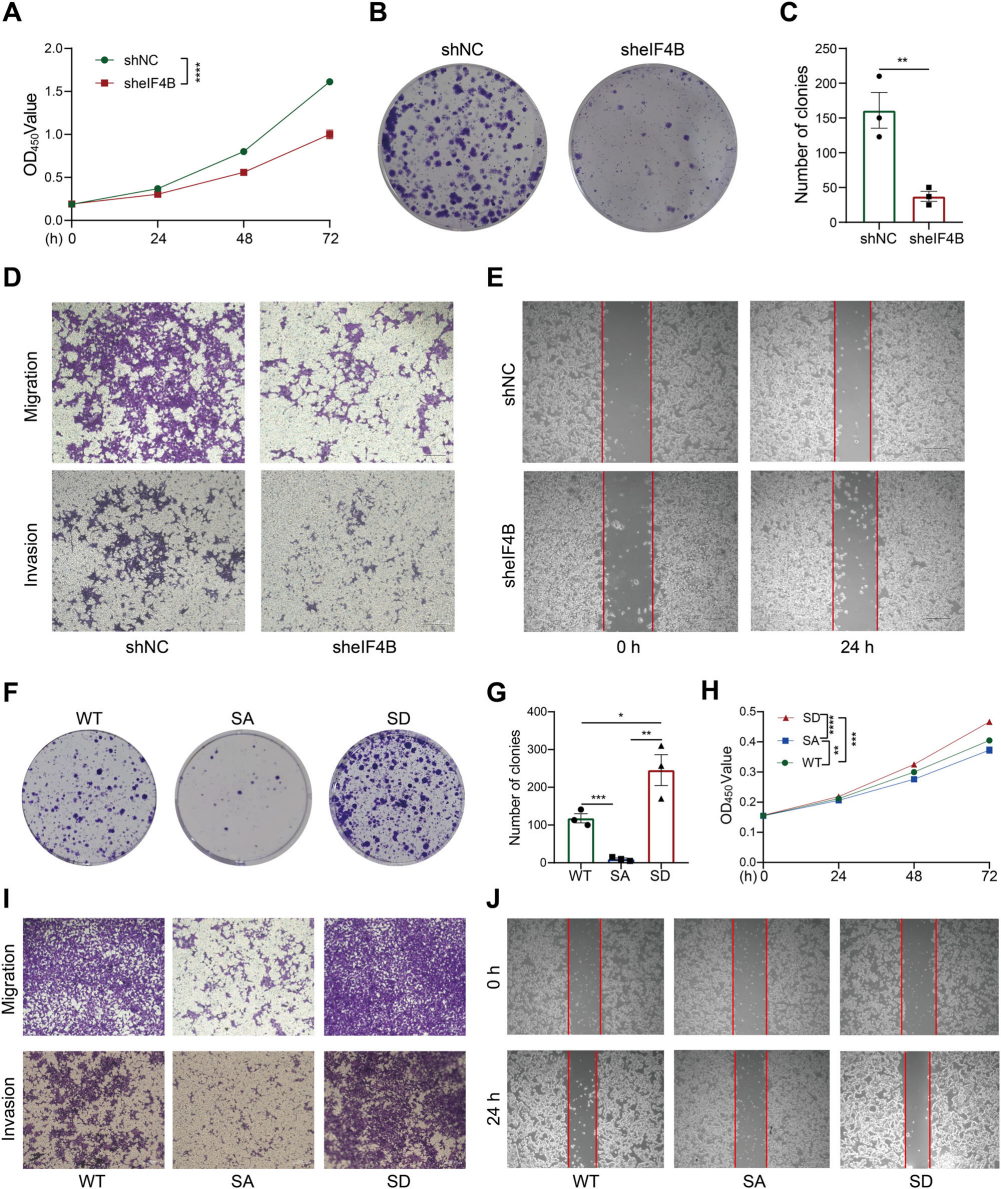
eIF4B knockdown inhibited CRC cell proliferation, migration, and invasion,while its Ser93 phosphorylation promoted these abilities in vitro. The following assays (**A**–**E**) were all conducted in the negative control (NC) and eIF4B knockdown cells. **A** CCK-8 assays were used to evaluate cells viability. **B**, **C** Colony formation assays were conducted to assess the proliferation capacity. Representative images and quantification of colonies were presented. **D** Transwell migration and invasion assays were conducted to evaluate cell migratory and invasive abilities. Representative images were presented with scale bar as 100 μm. **E** Wound‑healing assays were conducted to evaluate cell migratory abilities. Representative images were presented with scale bar as 200 μm. The assays of (**F**–**J**) were performed in eIF4B WT, eIF4B S93A, and eIF4B S93D CRC cells. **F**, **G** Colony formation assays were used to detect cell proliferation capacities. Representative images and quantification of colonies were presented. **H** CCK-8 assays were conducted to evaluate cell viabilities. **I** Transwell migration and invasion assays were conducted to evaluate the cell migratory and invasive abilities. Representative images were presented with scale bar as 100 μm. **J** Wound‑healing assays were conducted to evaluate cell migratory abilities. Representative images were presented with scale bar as 200 μm. Error bars represent mean±SEM from at least three independent experiments. *P* values were determined by two-sided Student t test. *P < 0.05, **P < 0.01, ***P < 0.001, and ****P < 0.0001.

Having established that both eIF4B and its phosphorylation at Ser93 are upregulated in CRC, we next investigated their biological functions in CRC cells. The eIF4B stable knockdown HT29 cell (sheIF4B HT29) and the control (shNC HT29) were generated (Fig. S2). The lack of eIF4B decreased the viability and proliferation ability of CRC cells proving by the CCK8 and anchorage-dependent colony formation experiments, respectively (Fig. 3A–C). In the Transwell migration and wound healing assay, eIF4B deficiency suppressed cell migration compared to shNC cells (Figs. 3D, E and S3A, B). Similar to the migration ability, the invasion ability of CRC cells was also attenuated by eIF4B knockdown (Figs. 3D and S3A). To further explore the function of eIF4B Ser93 phosphorylation in CRC, we stably re-expressed eIF4B WT, S93A, and S93D mutant in the sheIF4B HT29 cells. In anchorage-dependent colony formation and CCK8 assays, eIF4B S93A mutant decreased while eIF4B S93D mutant increased CRC cells viability and proliferation compared to eIF4B WT cells (Fig. 3F–H). Furthermore, re-expression of eIF4B S93D mutant promoted cell migration and invasion compared to re-expression eIF4B WT, as determined by Transwell assay and wound healing. In contrast, re-expressing eIF4B S93A mutant hardly restored the migration and invasion of sheIF4B HT29 cells (Figs. 3I, J and S4A, B). In conclusion, we found that knockdown eIF4B decreased CRC cells proliferation, migration and invasion. Moreover, eIF4B Ser93 phosphorylation was essential for the proliferation, migration and invasion of CRC cells.

### eIF4B Ser93 phosphorylation increased the translation of mesenchymal makers through enhancing eIF4B protein stability and translation activity

**Fig. 4:**
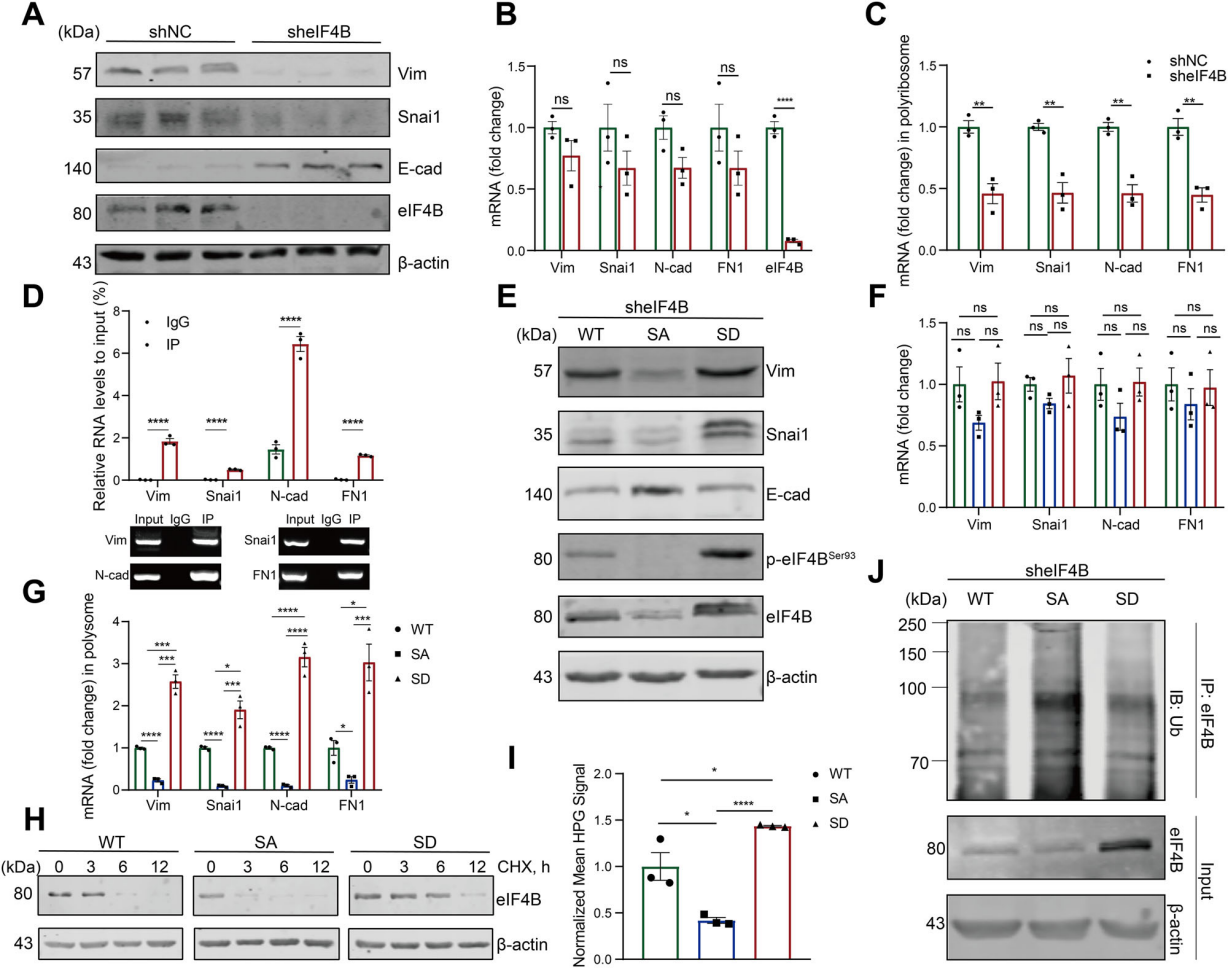
eIF4B Ser93 phosphorylation boosts mesenchymal markers translation by enhancing eIF4B translational activity and stability. **A** Western blotting of EMT markers (Vim, Snai1, and E-cad) in NC and eIF4B knockdown cells. **B** The levels of mesenchymal mRNA (N-cad, Vim, Snai1 and FN1) were measured by RT‒qPCR. **C** RT-qPCR analysis of the mRNA abundance of mesenchymal markers in polyribosome fractions collected by sucrose gradient centrifugation in shNC and sheIF4B CRC cells. **D** Cell lysates prepared from HT29 cells were subject to immunoprecipitation (IP) with antibodies against IgG and eIF4B, followed by RT-qPCR with primers specific for N-cad, Vim, Snai1 and FN1. Verification of eIF4B binding on N-cad, Vim, Snai1 and FN1 mRNA by agarose gel electrophoresis analysis of the qPCR products. The assays of (**E**–**J**) were performed in eIF4B WT, eIF4B S93A, and eIF4B S93D CRC cells, respectively. **E** Western blotting analysis of EMT markers. **F** The levels of mesenchymal mRNA were measured by RT‒qPCR. **G** RT-qPCR analysis of the mRNA abundance of mesenchymal markers in polyribosome fractions collected by sucrose gradient centrifugation. **H** The CHX pulse-chase assay of eIF4B was performed to detect the protein stability of eIF4B. **I** Protein synthesis assays showing relative levels of newly synthesized proteins. **J** The cells were treated with MG132 for 6 h, and then followed by ubiquitination assay performed with anti-eIF4B IP and anti-ubiquitin IB analyses. All the data are presented as mean±SEM from at least three independent experiments. *P* values were determined by two-sided Student t test. *P < 0.05, **P < 0.01, ***P < 0.001, and ****P < 0.0001.

We have found that the alteration of protein phosphorylation related to cell migration, adhesion and EMT in CRC tissues. To study the mechanisms involved in eIF4B-regulated CRC cell function, we detected EMT signaling in CRC cell lines. Western blotting showed that eIF4B deficiency significantly decreased the protein levels of mesenchymal makers, such as Vim (Vimentin) and Snai1 (Snail 1), while increased the protein level of epithelial marker E-cad (E-cadherin) (Fig. 4A). Strangely, RT-qPCR showed that the mRNA levels of mesenchymal makers including Vim, Snai1, N-cad (N-cadherin), and FN1 (Fibronectin 1) had no difference between eIF4B knockdown and the control cells (Fig. 4B). Given that eIF4B is the important initiation factor for mRNA translation, we performed sucrose gradient centrifugation to acquire polysome to quantify mRNA in a translation state. The polysome-bound mRNA levels of mesenchymal markers including Vim, Snai1, N-cad, and FN1 were significantly decreased in eIF4B knockdown cells (Fig. 4C). We further found that eIF4B was able to bound to the mRNA of these mesenchymal markers (Fig. 4D), indicating that eIF4B knockdown decreased mesenchymal makers by reducing their mRNA translation.

Our previous results indicates that eIF4B Ser93 phosphorylation is crucial for CRC malignant function. Based on the LinkedOmics analysis, the phosphorylation of eIF4B Ser93 was found to be significantly (*p* < 0.05) positively correlated with the expression of mesenchymal genes, such as Twist1, MMP3, and MMP1, while negatively correlated with the expression of epithelial gene, CDH1 (E-cad) in CRC patients (Fig. S5). Western blotting indicated that, in comparison to eIF4B WT cells, the protein levels of Vim and Snail increased, whereas the protein of E-cad decreased in eIF4B S93D cells. Conversely, in eIF4B S93A cells, the expression of EMT markers exhibited an opposite trend (Fig. 4E). Similar to eIF4B knockdown, eIF4B Ser93 phosphorylation had no effect on mesenchymal markers mRNA levels (Fig. 4F). Moreover, re-expression of eIF4B S93D mutant in sheIF4B HT29 cells resulted in a significantly higher translation of mesenchymal markers mRNA compared to eIF4B WT. In contrast, the eIF4B S93A mutant did not restore the translation levels compared to eIF4B WT (Fig. 4G), indicating that phosphorylation of eIF4B at Ser93 is indispensable for its role in the translation of mesenchymal markers. However, the role of eIF4B Ser93 phosphorylation in eIF4B regulation was unclear. CHX pulse-chase assay showed that eIF4B S93A mutant accelerated eIF4B protein degradation while eIF4B S93D mutant made eIF4B more stable (Figs. 4H and S6). Furthermore, eIF4B S93D mutant activated eIF4B translation activity and increased proteins synthesis, while eIF4B S93A mutant inhibited this process (Fig. 4I). In vivo ubiquitination assay further revealed that eIF4B S93A mutant increased the ubiquitination of eIF4B, while eIF4B S93D mutant decreased eIF4B ubiquitination, indicating that eIF4B Ser93 phosphorylation might enhance eIF4B protein stability by reducing its ubiquitination (Fig. 4J). Taken together, we proved that eIF4B S93 phosphorylation increased eIF4B protein stability and translation activity through which increased the translation of mesenchymal makers.

### eIF4B knockdown suppressed the growth and metastasis of CRC, while eIF4B Ser93 phosphorylation promoted these processes in vivo

**Fig. 5:**
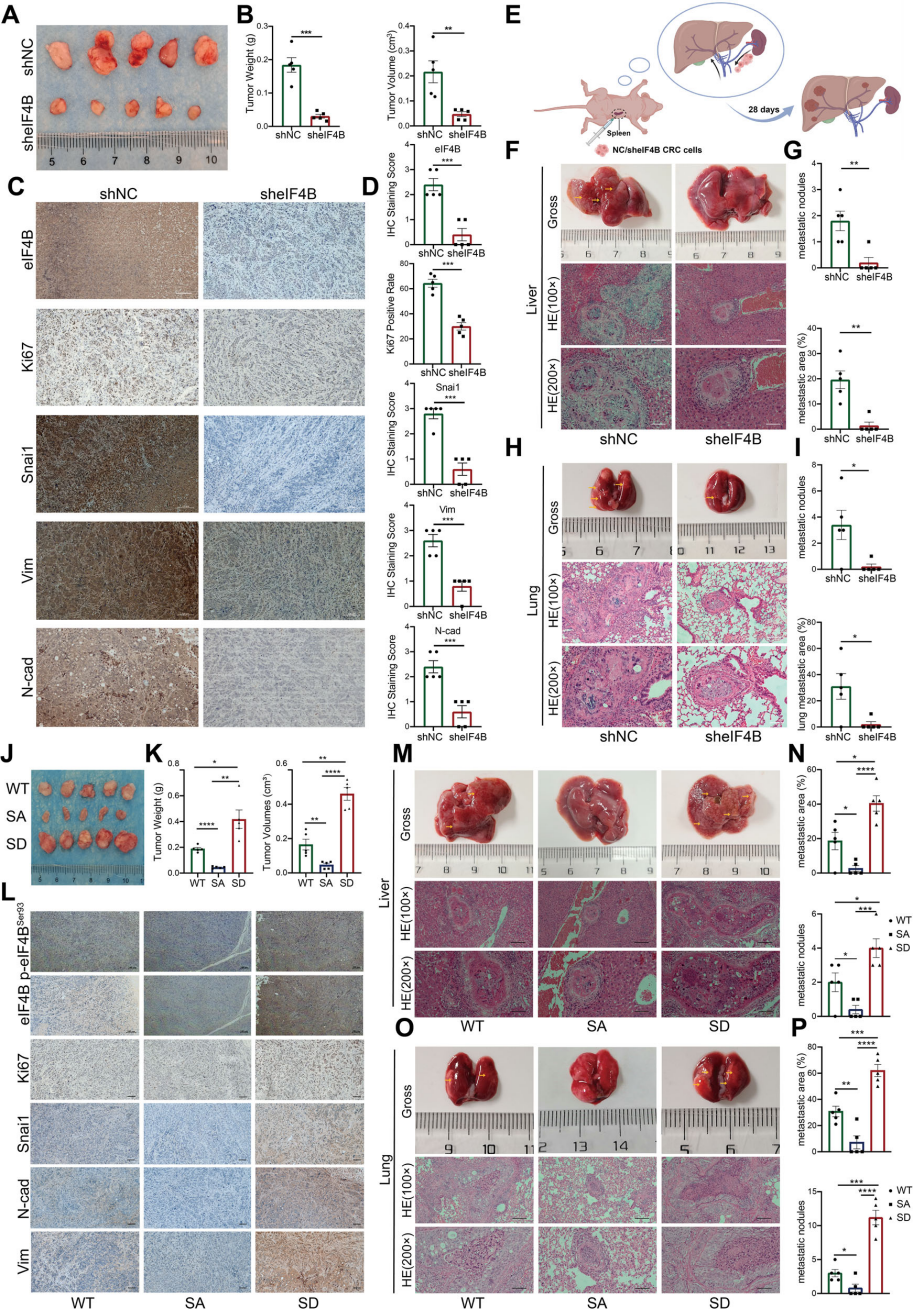
eIF4B knockdown inhibited CRC growth and metastasis, while Ser93 phosphorylation promoted these processes in vivo. **A** The subcutaneous tumors generated from shNC and sheIF4B CRC cells were dissected and photographed. **B** Tumor weight and volume were recorded and analyzed. **C** Immunohistochemistry was used to visualize and compare the protein levels of eIF4B, Ki67, Snai1, Vim and N-cad in tumors collected from the two groups. Scale bar: 100 μm. **D** The xenograft IHC staining score of eIF4B, Snai1, Vim and N-cad, and the staining positive rate of Ki67. **E** Schematic illustration of splenic injection-induced liver metastasis model in nude mice. **F** Twenty-eight days later, mice were sacrificed, and liver tissues were photographed and subjected to H&E staining. **G** The number of liver metastatic nodules and area were counted. **H** Lung tissues collected from lung metastasis model via tail vein injection in nude mice were photographed and subjected to H&E staining. **I** The number of lung metastatic nodules and area were counted. The eIF4B WT, eIF4B S93A, and eIF4B S93D CRC cells were subcutaneously injected into the abdominal flanks of nude mice, respectively. Gross images, tumor volumes and tumor weights of these three groups were shown in (**J**) and (**K**). **L** Immunohistochemistry was used to visualize and compare the protein levels of eIF4B, p-eIF4B^Ser93^, Ki67, Snai1, Vim and N-cad in tumors collected from the three groups. Scale bar: 100 μm. **M** The tissues of liver metastasis model generated from the three groups were photographed and subjected to H&E staining. **N** The number of liver metastatic nodules and area were counted. **O** Lung tissues were collected, photographed, and subjected to H&E staining after the establishment of lung metastasis model. **P** The number of lung metastatic nodules and area were counted. All the data are presented as mean±SEM (*n* = 5). *P* values were determined by two-sided Student t test. *P < 0.05, **P < 0.01, ***P < 0.001, and ****P < 0.0001.

Having established the significant role of eIF4B Ser93 phosphorylation in promoting malignant phenotypes of CRC cells in vitro, we therefore sought to validate the function of eIF4B in vivo. To this end, we established subcutaneous xenograft models in nude mice to assess its impact on tumor growth (Fig. S7A). The weight and volume of xenograft tumors in the eIF4B-deficient groups were significantly reduced compared to those in the control group (Fig. 5A, B). A similar result was observed by IHC staining of proliferation marker Ki-67 (Fig. 5C, D). Furthermore, the expression levels of mesenchymal markers, including Snai1, Vim, and N-cad, were all decreased in eIF4B-deficient xenograft tissues (Fig. 5C, D). To further elucidate the function of eIF4B in CRC metastasis in vivo, we used a liver metastasis model via intrasplenic injection (Fig. 5E). EIF4B deficiency resulted in fewer liver metastatic nodules compared to the control group, confirmed by photograph and HE staining, which showed a significant reduction in both the number and area of metastatic nodules (Fig. 5F, G). Moreover, we also used a lung metastasis model established through tail vein injection to further verified the pro-metastasis role of eIF4B (Fig. S7B). Consistent with the liver metastasis findings, eIF4B deficiency also significantly reduced lung metastasis. Gross imaging and H&E staining revealed fewer metastatic nodules and a smaller total metastatic area in the lungs. (Fig. 5H, I).

The eIF4B WT, S93A, and S93D mutants were re-expressed in sheIF4B HT29 cells, respectively, and subsequently hypodermically injected into nude mice to further investigate the role of eIF4B Ser93 phosphorylation in vivo. The eIF4B S93D mutant was found to promote tumor growth, while the eIF4B S93A mutant inhibited tumor growth when compared to the eIF4B WT group, as evidenced by measurements of tumor weight and volume (Fig. 5J, K). Immunohistochemical analysis revealed that the expression levels of the proliferation marker Ki67 and mesenchymal markers increased in the xenograft tumors of S93D re-expression group, whereas these markers decreased in the S93A group compared to the WT group (Fig. 5L). To define eIF4B Ser93 phosphorylation function in CRC metastasis in vivo, re-expressed eIF4B WT, S93A, and S93D sheIF4B HT29 cells were injected into nude mice via the spleen and tail vein, respectively, to conduct CRC metastasis model. Re-expression eIF4B S93D mutant significantly increased the metastatic potential of CRC, with larger metastatic areas and more metastatic nodules in liver and lung compared to eIF4B WT. Conversely, the eIF4B S93A mutant reduced CRC metastatic potential, indicting by the smaller metastatic areas and fewer metastatic nodules in liver and lung (Fig. 5M–P). Summarily, the phosphorylation of eIF4B Ser93 is essential for CRC growth and metastasis in vivo.

### ERK2 directly binds and phosphorylates eIF4B Ser93 through which promotes mesenchymal markers translation

**Fig. 6:**
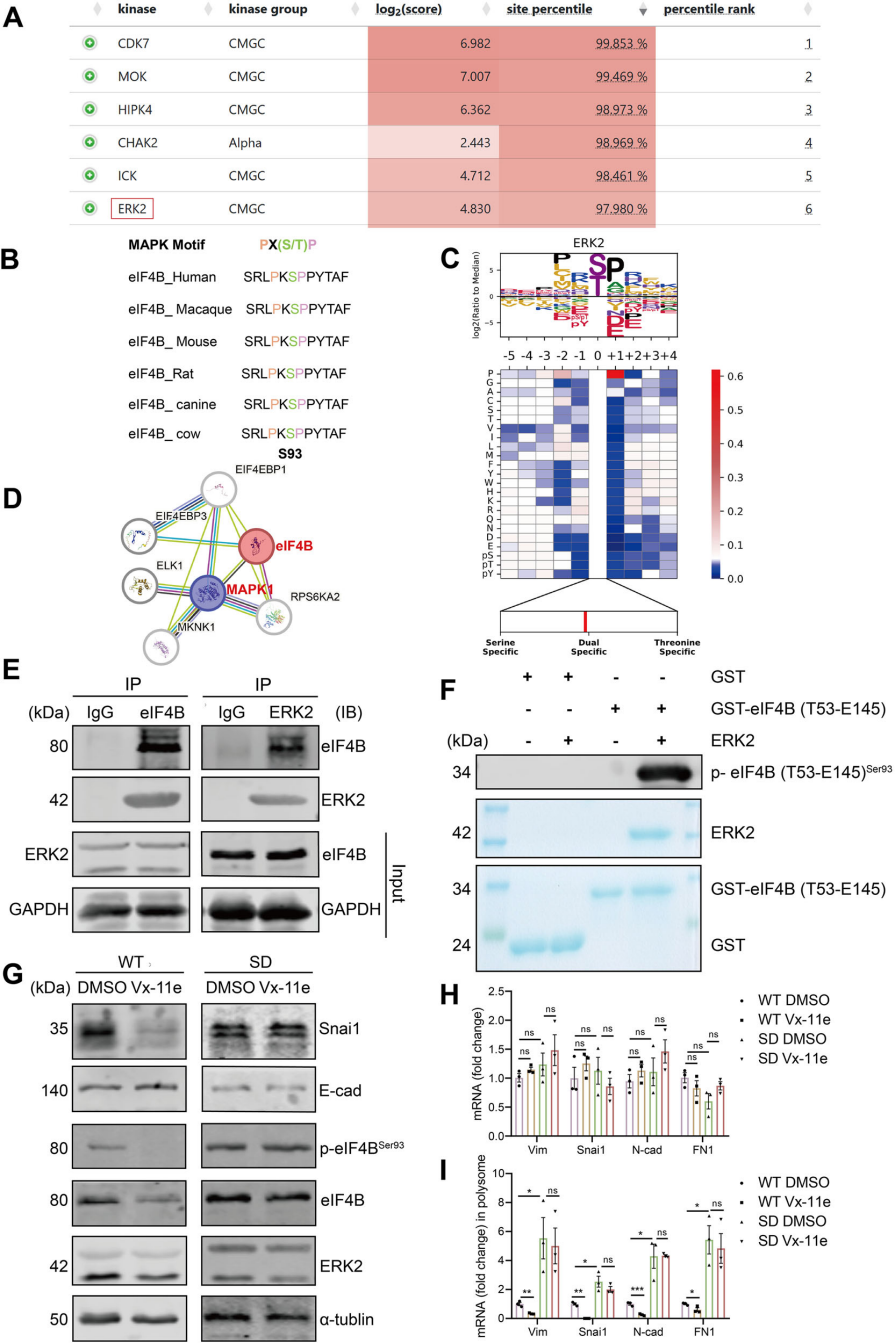
ERK2 directly binds and phosphorylates eIF4B Ser93 by which promotes mesenchymal markers translation. **A** The top six kinase which might regulate eIF4B Ser93 phosphorylation predicted by the “Kinase Prediction” website (https://www.phosphosite.org/kinaseLibraryAction) were shown. **B** The conserved amino acid sequence surrounding eIF4B S93 matches the consensus MAPK phosphorylation motif. **C** ERK2 was predicted as the kinase that regulated eIF4B Ser93 phosphorylation. **D** PPI network diagram of ERK2 (MAPK1) and eIF4B. **E** HT29 cell lysates were prepared by RIPA lysis and co-immunoprecipitated by ERK2 or eIF4B antibody with IgG as negative control. The immunoprecipitated protein was detected by western blotting. **F** GST-pull down assay showed the interaction between His-ERK2 and GST-eIF4B (T53-E145) protein in vitro. In vitro kinase assay and western blotting analysis of p-eIF4B (T53-E145)^Ser93^. The following assays were conducted in eIF4B WT and eIF4B S93D CRC cells following Vx-11e treatment (1 μM) or the control (DMSO) for 24 h. **G** The phosphorylation of eIF4B Ser93, eIF4B and EMT markers (Snai1, E-cad) protein were detected by western blotting. **H** The levels of mesenchymal makers mRNA were measured by RT‒qPCR. **I** RT-qPCR analysis of the mRNA abundance of mesenchymal markers in polyribosome fractions. All the data are presented as mean ± SEM from at least three independent experiments. *P* values were determined by two-sided Student t test. *P < 0.05, **P < 0.01, ***P < 0.001, and ****P < 0.0001.

To further explore the upstream regulation mechanism of eIF4B Ser93 phosphorylation, we applied “Kinase Prediction” website (www.phosphosite.org) to find the potential kinase that was able to phosphorylate S93 site motif “DRSRLPKS*PPYTAFL”. The kinases which comprehensive score ranked high were showed in Fig. 6A. Moreover, we applied the official website of “Cell signaling technology” (www.cellsignal.cn) to find the substrate consensus motif of these kinases. The amino acid sequence surrounding eIF4B-Ser93 site were evolutionally conserved and highly consistent with the predicted substrate motif of ERK2 and exactly matched the consensus MAPK (include ERK2) phosphorylation motif——PX(S/T)P, suggesting that ERK2 might directly phosphorylate eIF4B at Ser93 site (Fig. 6B, C). Moreover, PPI network analysis showed the interaction between MAPK1 (gene name of ERK2) and eIF4B (Fig. 6D).

We further confirmed the endogenous interaction between eIF4B and ERK2 in HT29 cells by co-IP (Fig. 6E). Moreover, glutathione S-transferase (GST)- pull down assay showed that ERK2 bound to eIF4B directly. To determine whether ERK2 directly phosphorylates eIF4B, in vitro kinase assay was performed using His-ERK2 and purified GST-eIF4B (T53-E145) as a substrate. Recombinant His-ERK2 phosphorylated GST-eIF4B (T53-E145) but not GST (Fig. 6F). To explore whether ERK2 regulated EMT through eIF4B Ser93 phosphorylation, we treated eIF4B WT and S93D CRC cells with ERK2 inhibitor Vx-11e. As shown in Fig. 6G, eIF4B Ser 93 phosphorylation and eIF4B protein levels decreased in eIF4B WT CRC cells after Vx-11e treatment. In contrast, Vx-11e did not affect eIF4B Ser93 phosphorylation or protein levels in eIF4B S93D CRC cells (Fig. 6G). Additionally, Vx-11e reduced the expression of mesenchymal markers in eIF4B WT CRC cells, while no such effect was observed in eIF4B S93D CRC cells (Fig. 6G). Furthermore, Vx-11e did not influence the mRNA levels of mesenchymal markers in either eIF4B WT or eIF4B S93D CRC cells (Fig. 6H). However, Vx-11e suppressed the translation of mesenchymal markers in eIF4B WT CRC cells, but not in eIF4B S93D CRC cells (Fig. 6I). In summary, these experiments demonstrate that ERK2 facilitates mesenchymal markers translation through directly binding and phosphorylating eIF4B Ser93.

### Vx-11e suppressed the proliferation, migration and invasion of CRC through eIF4B Ser93 phosphorylation inhibition

**Fig. 7:**
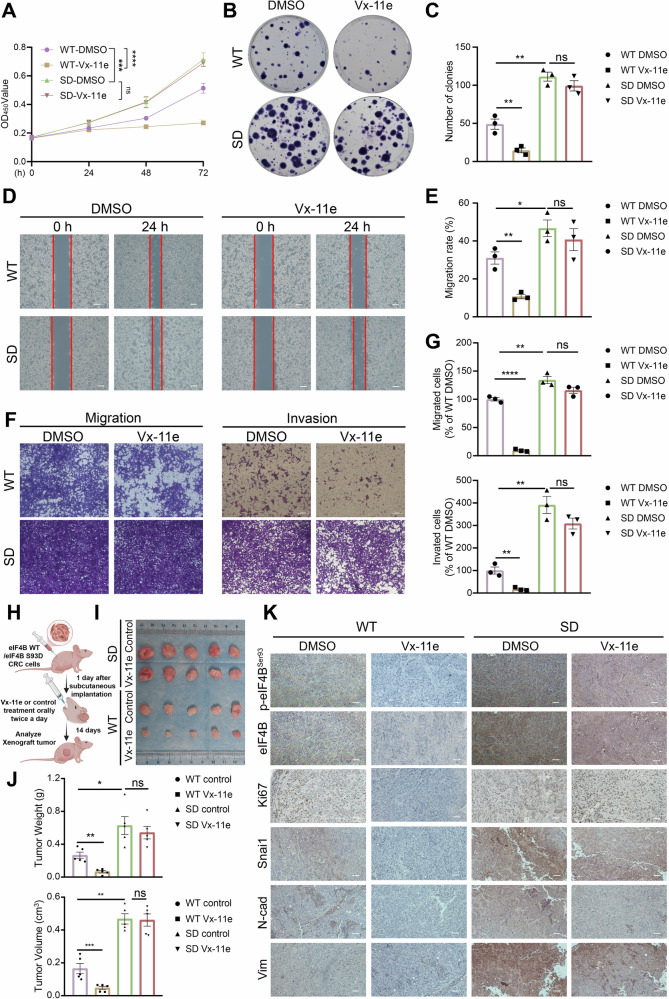
Vx-11e suppressed CRC progression by the inhibition of eIF4B Ser93 phosphorylation. The following assays were performed in eIF4B WT and eIF4B S93D CRC cells treated with Vx-11e (1 μM) or the control (DMSO). **A** CCK-8 assays were conducted to evaluate cells viabilities. **B**, **C** Colony formation assays were used to assess the cells proliferation capacities. Representative images and quantification of colonies were presented. **D**, **E** The cell migratory abilities were evaluated by wound‑healing assays. Representative images and quantification of wound closure are presented. Scale bar, 200 μm. **F**, **G** Transwell migration and invasion assays were conducted to measure cell migratory and invasive abilities. The number of migrated cells and invaded cells were presented as normalized values. Representative images were presented with scale bar as 100 μm. All the data are presented as mean±SEM from at least three independent experiments. **H** Schematic diagram of the xenograft tumor model following Vx-11e (50 mg/kg, twice a day) or the control (drug solvent without Vx-11e) treatment for 14 days established in nude mice with eIF4B WT or eIF4B S93D cells. **I**, **J** At day 14 after treatments, all mice were sacrificed, and tumors were dissected and photographed. Tumor weights and volumes were recorded and analyzed. **K** Immunohistochemistry was used to visualize and compare the level of p-eIF4B^Ser93^, and the protein levels of eIF4B, Ki67, Snai1, Vim and N-cad in tumors. Scale bar: 100 μm. All the data are presented as mean±SEM (*n* = 5). *P* values were determined by two-sided Student t test. *P < 0.05, **P < 0.01, ***P < 0.001, and ****P < 0.0001.

Finally, we further verified the role of Vx-11e in CRC progression both in vitro and vivo. eIF4B WT and eIF4B S93D CRC cells were treated with Vx-11e to investigate the role of Vx-11e in CRC function. CCK8 assay and anchorage-dependent colony formation indicated that Vx-11e inhibited the proliferation of eIF4B WT CRC cells. However, this anti-proliferative effect was not observed in eIF4B S93D mutant cells (Fig. 7A–C). Additionally, wound healing and Transwell assays demonstrated that Vx-11e effectively suppressed cell migration and invasion in eIF4B WT CRC cells, while exhibiting no significant effect in eIF4B S93D CRC cells (Fig. 7D–G). We further employed subcutaneous xenograft models in vivo (Fig. 7H), and observed that in the eIF4B WT group Vx-11e effectively suppressed CRC formation. Conversely, in the S93D group, Vx-11e exhibited little suppression of CRC growth (Fig. 7I, J). Additionally, IHC analysis revealed that Vx-11e decreased the levels of Ki67 and mesenchymal markers in eIF4B WT xenograft tissues (Fig. 7K). However, this down regulatory effect was not observed in the S93D group (Fig. 7K). Taken together, eIF4B Ser93 phosphorylation is essential for Vx-11e to suppress CRC growth and EMT both in vitro and vivo.

## Discussion

Researchers proved that aberrant alterations of protein phosphorylation have significant implications in CRC progression [29–31]. In this study, we performed a quantitative phosphorylation proteomics analysis of clinical CRC samples and controls. The results analyzed by bioinformatic method revealed that DPPs are linked to cell motility, migration, and EMT, with eIF4B Ser93 phosphorylation exhibited the most significant increase. Moreover, we further confirmed the phosphorylation of eIF4B Ser93 increased significantly in both patient tissues and CRC cell lines by a new created antibody specific targeting eIF4B Ser93 phosphorylation. Functionally, the increase eIF4B Ser93 phosphorylation promoted EMT and CRC cell proliferation and metastasis both in vitro and vivo. Mechanistically, the phosphorylation of eIF4B Ser93 increased its protein stability and translation activity, and thus promoting the translation of mesenchymal markers. Furthermore, ERK2 was identified as the upstream kinase which phosphorylates eIF4B at Ser93, and its inhibitor Vx-11e significantly diminished CRC proliferation and metastasis through decreasing eIF4B Ser93 phosphorylation. Collectively, our findings provide eIF4B Ser93 as a promising biomarker and target for CRC treatment.

Numerous studies have proved that eIF4B participant in various tumorigenesis [32–35]. The binding of eIF4B with histone mRNA facilitates tumor cell proliferation through promoting the smooth progress of S phase in diffuse large B-cell lymphoma [36]. EIF4B is crucial for anti-apoptosis in acute leukemia cells by regulating Bcl-2 and Bcl-XL expression [37]. Moreover, eIF4B interacts with FAM99A to affect the glycolytic metabolism of hepatocellular carcinoma [38]. However, the function of eIF4B in CRC remains elusive. In this study, we found that eIF4B knockdown significantly reduced the translation of mesenchymal markers such as Vimentin and Snai1. Interestingly, however, the protein level of E-cadherin was upregulated upon eIF4B depletion. We hypothesize that this upregulation is indirectly mediated through diminished translation of Snail, a known transcriptional repressor of E-cadherin[39]. As shown in Fig. 4C, eIF4B knockdown led to decreased Snail translation, which may alleviate transcriptional repression of the CDH1 gene, thereby increasing E-cadherin expression. This result underscores the role of eIF4B in regulating EMT through both translational and indirect transcriptional mechanisms. Functionally, eIF4B knockdown inhibited CRC growth and metastasis both in vitro and vivo. These results indicate that eIF4B may affect CRC progression through regulating EMT.

Different sites phosphorylation of eIF4B participants in different cell function in cancers. EIF4B Ser406 phosphorylation was reported to enhance breast cancer cell survival and proliferation by inhibiting anti-apoptosis [33], while Ser422 phosphorylation promotes CRC cell proliferation by regulating c-MYC synthesis [40]. In CRC liver metastases, eIF4B Ser422 phosphorylation has been shown to protect the survival and growth of CRC cells that have colonized in liver by enhancing ribosomal activity and increasing the synthesis of proteins associated with cell cycle and anti-apoptosis [41]. Although this study elucidates the significant impact of eIF4B Ser422 phosphorylation on the reactivation and proliferation of metastatic CRC cells in distant organs, the role of eIF4B phosphorylation in CRC metastasis has not been thoroughly investigated. Our findings reveal that the synthesis of mesenchymal markers is lower in CRC cells with eIF4B S93A mutation than in those with eIF4B WT. Conversely, CRC cells with eIF4B S93D mutation show increased synthesis of mesenchymal markers indicating eIF4B Ser93 phosphorylation promoted CRC cells EMT through mesenchymal makers translation. Furthermore, re-expressing eIF4B S93D significantly enhanced the growth and metastatic potential of CRC cells compared to re-expressing eIF4B WT. In contrast, eIF4B S93A did not restore these capabilities, highlighting the importance of eIF4B Ser93 phosphorylation in CRC cell proliferation and metastasis. In vivo experiments including liver/lung metastasis model also proved that eIF4B Ser93 phosphorylation promoted CRC distant metastasis. Although we have elucidated the function and mechanism of eIF4B Ser93 phosphorylation in CRC progression, additional research is required to determine whether other phosphorylation sites on eIF4B involved in CRC progression.

Phosphorylation of eIF4B typically enhances the translational efficiency of downstream target genes by increasing eIF4B’s activity [18]. Specifically, phosphorylation of eIF4B at Ser422 enhances the recruitment of 40S ribosomal subunit to eIF3 complex [41, 42]. Meanwhile, Ser406 phosphorylation boosts eIF4A’s helicase activity, aiding 40S small subunit’s recruitment to the initiation codon [43, 44]. However, whether eIF4B phosphorylation contributes to its own stability remained largely unknown. Intriguingly, we observed that increased eIF4B Ser93 phosphorylation in clinical CRC samples and cell lines was accompanied by a rise in total eIF4B protein. As demonstrated in Fig. 4H, J, the phospho-mimetic S93D mutant decreased eIF4B ubiquitination and prolonged its half-life, whereas the non-phosphorylatable S93A mutant had the opposite effect. Therefore, eIF4B Ser93 phosphorylation might be able to shield eIF4B from ubiquitination-mediated degradation and subsequently accumulate the eIF4B protein pool for eIF4B’s translational activation. This mechanism ensures a high level of functionally active eIF4B, which is critical for driving the efficient translation of mesenchymal markers to facilitate EMT and metastasis. Summarily, our data strongly suggest that post-translational stabilization is a key contributor to its protein upregulation in CRC.

ERK2 is a conserved core component of MAPK/ERK pathway and essential for cells survival, proliferation and migration [42–44]. In LAM, ERK2 activation increased Fra1 transcription, while the mTORC1/S6K1 pathway enhances Fra1 mRNA translation by phosphorylating eIF4B at Ser422 [22]. Whether ERK2 directly interacts with eIF4B to affect tumor progression is unclear. We identify ERK2 as the upstream kinase of eIF4B which directly binds and phosphorylates eIF4B at Ser93. Inhibition of ERK2 by Vx-11e suppressed the phosphorylation of eIF4B Ser93 as well as mesenchymal markers translation. Vx-11e is a potent ERK2 inhibitor with significant efficacy in tumor therapies [45–47]. Combining it with the PI3K inhibitor BKM120 effectively inhibits the growth of BRAF inhibitor-resistant melanoma in xenograft models. This therapy has promised to be included in second-line treatments for patients resistant to vemurafenib and dabrafenib [48]. We found that Vx-11e suppressed eIF4B Ser93 phosphorylation through which suppressed mesenchymal marker translation. Functionally, Vx-11e decreased CRC cell proliferation and migration, but was ineffective in eIF4B S93D mutant cells. In vivo experiments also showed that Vx-11e suppressed CRC progression and metastasis, but was ineffective in eIF4B S93D mutant CRC xenograft model, which suggested that inhibiting eIF4B Ser93 phosphorylation is essential for Vx-11e’s anti-tumor effects in CRC. However, clinical application of Vx-11e for CRC treatment requires further studies.

In this study, we identified eIF4B Ser93 phosphorylation as a crucial intermediary in CRC EMT and metastasis. ERK2 was confirmed as the upstream kinase that directly promotes eIF4B Ser93 phosphorylation, thereby providing a theoretical basis for the clinical application of ERK2 inhibitor Vx-11e in CRC treatment. While the upstream regulation of the ERK2/eIF4B Ser93 axis is not fully elucidated, the gut microbiota represents a compelling candidate [49]. Given its established role in regulating inflammation, immunity, and cellular signaling [50–52], we hypothesize that microbial-derived factors may activate ERK2, thereby initiating the eIF4B Ser93 phosphorylation cascade that drives EMT. This perspective positions the ERK2/eIF4B axis as a molecular bridge converting microbial signals into pro-metastatic translational output. Additionally, whether ERK2/eIF4B Ser93 axis affect gut microbiota homeostasis through EMT through which forming a positive feedback loop need further study. Future studies are warranted to investigate how specific microbiome components or metabolites regulate this pathway.

Beyond the ERK2/eIF4B Ser93 axis, our phosphoproteomic data reveal a broader network of phosphorylation alterations involved in CRC progression. Numerous DPPs—such as Zyxin (ZXY, Ser143) and Filamin A (FLNA, Ser2152)—show significantly increased phosphorylation and are closely linked to cell adhesion, migration, and EMT. Zyxin, a focal adhesion (FA) protein, has been reported to promotes tumor progression by modulating cytoskeletal reorganization and EMT [53]. Similarly, FLNA, an actin-crosslinking protein, is upregulated and phosphorylated at Ser2152 in Snail-overexpressing cells, where it stabilizes FA and regulates cell motility [54]. These findings collectively suggest that CRC metastasis is orchestrated by a complex phospho-regulatory network, in which the ERK2/eIF4B Ser93 axis represents one important, but not exclusive, pathway. Subsequent studies will delineate the functions and underlying mechanisms of these phosphoproteins to explore more novel targets for CRC therapy.

**Fig. 8:**
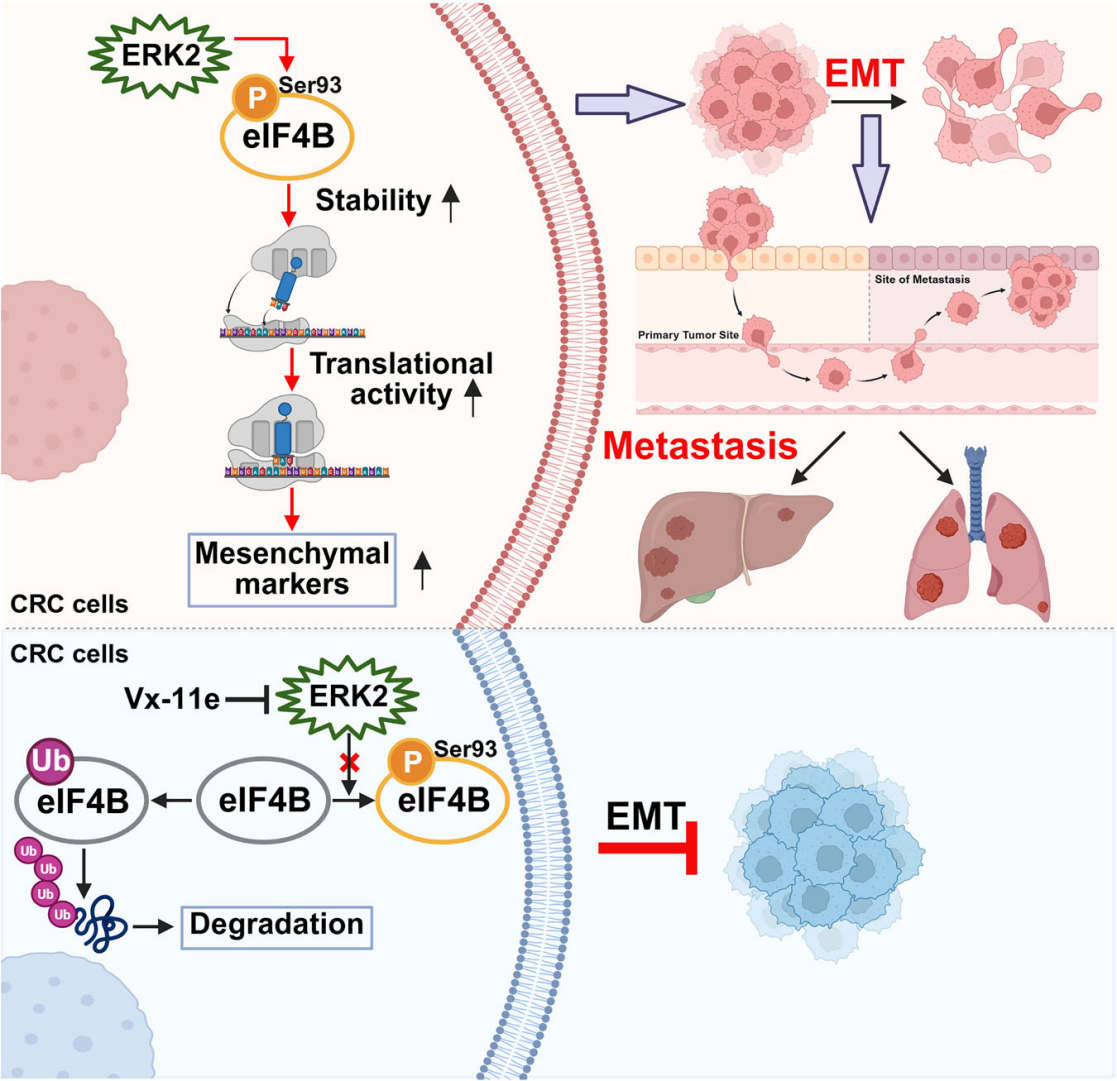
Schematic illustration of the current study. EIF4B Ser93 phosphorylation by ERK2 promotes mesenchymal markers translation to drive CRC metastasis, while inhibition of ERK2 by Vx-11e suppresses eIF4B phosphorylation at Ser93 site and facilitates eIF4B degradation by ubiquitination, and thus inhibiting EMT and tumor metastasis (Created with BioRender. com).

In conclusion, our study delineates a novel metastatic mechanism in CRC driven by ERK2-mediated eIF4B Ser93 phosphorylation, which enhances eIF4B protein stability and promotes the translation of mesenchymal markers (Fig. 8). These findings highlight the clinical potential of targeting this pathway, as the consistent elevation of eIF4B Ser93 phosphorylation in CRC positions it as a promising diagnostic and prognostic biomarker for metastatic risk assessment. Moreover, the ERK2 inhibitor Vx-11e effectively suppresses tumor growth and metastasis in preclinical models by specifically targeting eIF4B Ser93 phosphorylation, suggesting a viable therapeutic strategy for advanced CRC. Together, our results support further exploration of the ERK2/eIF4B-Ser93 pathway as both a biomarker and a therapeutic target in CRC clinical management.

## Materials and methods

### Patients and sample collection

Colorectal cancer tissue samples (including tumor tissue and normal tissue at least 5 cm from the tumor margins) were obtained from patients with a pathologically confirmed diagnosis of colorectal cancer who had not received any treatment. The use of clinical samples with patient consent in this study were approved by the Ethical Committee, Guangxi Medical University Cancer Hospital (Approval Number: KY2025637). And the xenograft tumorigenesis model, liver-metastatic model, and lung-metastatic model were approved by the Animal experimental center of Guangxi Medical University (Approval Number: 202306021). The operations and experimental protocols were performed according to the laboratory guidelines of the National Institutes of Health (NIH). Detailed information of the surgical samples is listed in Supplementary Material 1.

### TMT label-phosphoproteomic analysis

TMT labeling LC‒MS/MS assay to detect differentially expressed phosphoproteins (DPPs) in CRC and the control tissues was performed by Shanghai Applied Protein Technology Co., Ltd. Briefly, CRC protein samples (*n* = 30) were divided into three tumor samples, while the normal samples (*n* = 30) were divided into three control samples. An amount (100 μg) of trypsinic peptide mixture of each sample was labeled using TMT according to the manufacturer’s instructions (90066, Thermo Fisher Scientific). LC–MS/MS analysis of phosphopeptides was performed on a Q Exactive HF mass spectrometer (Thermo Scientific) that was coupled to Easy nLC (Proxeon Biosystems, now Thermo Fisher Scientific). MS/MS data were processed using MASCOT engine (Matrix Science, London, UK; version 2.2) embedded into Proteome Discoverer 2.4. Tandem mass spectra were searched against the human uniprot database (www.uniprot.org) concatenated with a reverse decoy database. Trypsin was specified as the cleavage enzyme, allowing up to 2 missing cleavages. Mass tolerance for precursor ions was set to 20 ppm in the initial search and 5 ppm in the main search. Mass tolerance for fragment ions was set to 0.1 Da. Cysteine carbamidomethylation was specified as a fixed modification, and oxidation and phosphorylation (ST)/ phosphorylation (Y) were specified as variable modifications. FDR was adjusted to <0.01 with a minimum score for modified peptides set to >60. The protein contained specific phosphopeptide meeting the following conditions were defined as DPPs: multiple changes (fold change) >1.2, *P* < 0.05.

### Cell culture

The normal human intestinal epithelial cell HIEC and CRC cell lines HT-29, HCT116, SW480, RKO, Lovo, and Caco-2 were purchased from the Pricella Biotechnology Co., Ltd (Wuhan, China). All cells were cultured in Dulbecco’s Modified Eagle Medium (DMEM, Gibco) supplemented with 10% fetal bovine serum (Vazyme) except for Caco-2, which was cultured in DMEM with 20% fetal bovine serum supplemented with recombinant human non-essential amino acid (NSAA, 100 ng/mL, MCE). Cells were maintained at 37 °C in a humidified atmosphere containing 5% CO_2_ and were revived every 3 to 4 months. All cell lines were verified by STR profiling and tested for mycoplasma contamination. ERK2 inhibitor Vx-11e (Selleck, S7709) were used in a final concentration as 1 μM in specific assays. All cell culture work was performed under aseptic conditions in a laminar flow biosafety cabinet (Class II, Type A2) following standard laboratory protocols.

### Anti-phospho-Ser93-eIF4B antibody generation

To generate antibody against phospho-Ser93-eIF4B, a 10–16 weeks process comprising six phases was conducted by ProteinGene Biotech Co., Ltd (Wuhan, China), as follows. Phase I included the design and synthesis of the Ser93-eIF4B peptide (1-3 weeks). Conjugated peptides, SRLPK(phospho-)SPPYTAC (88-98 aa), as immunizing sequence were then used to immunize rabbits through 4-6 rounds of immunization in phase II (8-12 weeks). The immunized blood (100-120 mL) was collected and prepared for antiserum (phase III). Then the potency of the antiserum was detected by ELISA assays in phase IV. Antibody was purified by procedures that included antigen affinity and special affinity purification (phase V). The resultant samples were suspended in PBS containing 0.03% sodium azide, and the specificity of each purified antibody was verified in phase VI.

### Immunohistochemistry and scoring

Samples of xenograft tumors were obtained from mice undergoing subcutaneous injection of shNC and sheIF4B HT29 cells or re-expressed eIF4B S93D, S93A and WT sheIF4B HT29 cells. The samples used for paraffin sections were fixed with 10% formalin immediately after dissection. The score of the staining intensity of stained tumor cells was assessed by the plug-in of Image J, IHC, profiler. Cells were scored according to the intensity of staining into 4 levels: negative scored 0; weakly positive scored 1; positive scored 2; and strongly positive scored 3. And all chemical operations were performed in a well-ventilated area with proper personal protective equipment.

### Western blotting

Protein samples were harvested by RIPA buffer (CWBIO, Taizhou) supplemented with a protease inhibitor cocktail and phosphatase inhibitors, and denatured by boiling for 10 min. Then the samples were separated based on size using 10% SDS-polyacrylamide gel electrophoresis. The transfer time was determined according to the size of the target protein, and then the interested proteins were transferred to NC membrane. The membrane was blocked with 5% BSA at room temperature for 1 h and incubated with corresponding primary antibody at 4 °C overnight. The next day, the membrane was incubated with IRDye^®^ secondary antibodies at room temperature for 1 h in the dark. Finally, the membranes were photographed by the Odyssey CLX Western Blotting imaging (Li-Cor, 1705061). The antibodies used in this study are listed in Supplementary Table 1.

### Lentivirus and the of stable cell lines generation

To knock down eIF4B in HT29 cells, GenePharma Co., Ltd. (Suzhou, China) designed and obtained DNA oligo targeted eIF4B and embedded it into the LV3 (H1/GFP&Puro) vector to generate sheIF4B lentivirus. To re-express eIF4B WT (wild type), S93A (inactive mutant), and S93D (active mutant) in sheIF4B HT29 cells, GenePharma designed and obtained cDNA containing full-length eIF4B, full-length eIF4B with S93A mutation and full-length eIF4B with S93D mutation and embedded it into the LV18C (LV18-CMV-T2A-Neo) vector, respectively, to generate according overexpressing lentivirus. For the generation of sheIF4B HT29 stable cell lines, we treated cells with puromycin (2 μg/ mL) to select the transfection cells, while sheIF4B cells were treated with G418 (200 μg/mL) to generate over-expressing stable cell lines.

#### CCK-8 assay

To evaluate cell viability, we employed Cell Counting Kit-8 (Vazyme, China) according to the manufacturer’s instructions. Briefly, 1 × 10^3^ pretreated cells were seeded into 96-well plates after counting, and the volume of medium added to each well was 100 μL. Every 24 h later, 10 μL CCK8 solution was added to corresponding wells, and the cells were incubated for 30 minutes in a 37 °C incubator. Finally, absorbance (450 nm) was measured by a SpectraMax^®^ iD5 reader.

### Colony formation assay

A total of 1 × 10^3^ cells were cultured in 6-well plates for colony formation. After two weeks of incubation, the cells were fixed with 4% paraformaldehyde and stained with crystal violet. The colonies were photographed and counted.

### Cell migration and invasion assays

For migration assay, equal numbers (3 × 10^4^) of cells were seeded into the upper chamber and incubated with serum-free medium. And then 500 μL complete medium supplemented with 10% FBS was added into the lower chamber of the Transwell insert to promote cell migration or invasion. For invasion assay, a Matrigel polycarbonate membrane (Corning) was placed in the upper chamber. After another 48 h incubation, cells migrating or invading through the membrane of Transwell inserts were stained with crystal violet and photographed by microscopy (100 ×).

### Wound healing assay

Cells from different treatments were cultured in 6-well plates until 90% confluence, and then scratches were created with a 200-μL pipette tip. Medium was replaced into the serum free to avoid the interference of cell proliferation. The bright-field images of the samples at 0 and 24 h after scratching were acquired with a microscope. ImageJ was used to calculate the migratory rate.

### RNA isolation and

#### quantitative real-time PCR analysis

Total RNA was extracted by TRIzol^®^ RNA Isolation Reagent (Invitrogen, USA). After reverse transcription by HiScript III qRT SuperMix (Vazyme, China), polymerase chain reaction was conducted by a Real-time Fluorescent Quantitative PCR system. β-actin was used as an endogenous control. The 2^−^^ΔΔCt^ method was used to calculate the relative expression level of target genes. The primers used in this study are listed in Supplementary Table 2.

### RNA immunoprecipitation (RIP)

About 1 × 10^7^cells were lysed by RIP lysis (1 M HEPES-KOH, 8 mg/mL NaCl, 0.5 M EDTA, 1%TritonX-100, 1 mg/mL deoxycholate, 1 mg/mL SDS). Normal rabbit IgG was used as the negative control. Protein A/G agarose was used to crosslink antibodies. Finally, the isolated RNA in the pulldown products were analyzed by RT-qPCR, and following by agarose gel electrophoresis.

### Sucrose gradient centrifugation and polysome fractionation

Briefly, cells were lysed in polysome cell extraction buffer (1 M MOPS, 1 M MgCl_2_, 5 M NaCl, 10 mg/ml CHX, 10% Triton X-100, 2 mg/ml heparin, 40 U RNaseOUT, 0.1 M PMSF, and 100 mM benzamine) on ice. Cellular debris was cleared by centrifugation at 13,000 × *g* for 10 min at 4 °C. Extracts were loaded on a 10–50% sucrose gradient and centrifuged at 32,000 rpm for 3 h at 4 °C in an SW 32 Ti rotor (Beckman coulter). Then RNA in the polysome fraction was extracted for RT-qPCR.

### CHX chase assay

CHX (10 μM) was added to the medium at 0, 3, 6, 9 and 12 h in advance according to the different time course. Cells were harvested and the total protein was extracted at the time point, and then the change of target protein was verified by Western Blotting.

### Co-immunoprecipitation

RIPA buffer supplemented with a protease inhibitor cocktail was employed to lyse the cells. The protein A/G magnetic beads (HY-K0202, MCE) were preprocessed with equal IgG controls and indicated antibodies according to the manufacturer’s instructions, and then were added into equal protein supernatant. Slowly rotate the mixture at 4 °C for 2 hours. And then, the precipitates (bead-antibody-protein complex) were washed with ice-cold PBST buffer 3 times. Precipitation was harvested by SDS loading and denatured by boiling for 5 min. Western blotting was used to detect the precipitated protein.

### GST-pulldown assay and in vitro kinase assay

Commercial recombinant human GST (HY-P70270, MCE), GST-eIF4B (T53-E145) (HY-P7S0844 MCE), and His-ERK2(HY-P75223, MCE) were subjected to GST pull-down assay. The conjugation of GST and GST-eIF4B (T53-E145) with anti-GST magnetic beads (HY-K0222, MCE) and the pull-down assay were performed according to the beads’ instructions. GST or GST-eIF4B (T53-E145) were incubated with activated His-ERK2 and 200 μM ATP (HY-B2176, MCE) in a kinase buffer (HY-K0016, MCE) at 30 °C for 90 minutes. The kinase reaction was stopped by the addition of SDS PAGE loading buffer and boiling, and subsequently subjected to Western blotting.

### Animal models

Cells (5 × 10^6^) were suspended in PBS and injected subcutaneously into nude mice (4 weeks old) to generated subcutaneous tumor model. After 7 days, the mice were euthanized by overdose narcotic drugs and tumors were harvested from sacrificed mice and weighed. The volume of the tumor was speculated by the formula below: tumor volume = 0.5 × length × width^2^ (mm^3^). Similarly, eIF4B WT and S93D stably re-expressed sheIF4B HT29 cells were injected subcutaneously. The next day after injection, animals were randomized into Vx-11e treatment groups (50 mg/kg, twice a day) or the control groups. Mice were sacrificed after two weeks of treatment or when necessary for animal welfare. The mice were euthanized by overdose narcotic drugs. The harvested tumors were weighed and measured volumes as above. Moreover, spleen injection-liver metastasis model was conducted. Six-week-old male nude mice were anesthetized using an intraperitoneal injection of avertin solution (Meilunbio). Under aseptic conditions, a small longitudinal incision was made in the left upper flank, and 2.5 × 10^6^ cells in 50 µl PBS were injected with a 30-gauge needle into the spleen capsule. Animals were sacrificed after 28 days, and the livers were dissected. In tail vein-lung metastasis model, cells (2.5 × 10^6^) were suspended in PBS and injected via the tail vein into 6-week-old nude mice. Twenty-eight days later, the mice were sacrificed, and the lung tissues were dissected. Tissues were fixed for H&E staining. During our study, the mice were kept under specific pathogen-free conditions with a 12 h light/dark cycle. The protocol was approved by the Animal experimental center of Guangxi Medical University.

### Bioinformatics analysis

The uniprot IDs of DPPs were analyzed by David database (https://davidbioinformatics.nih.gov) for Gene Ontology (GO) and Kyoto Encyclopedia of Genes and Genomes (KEGG) pathway analysis to identify their functions and enriched pathways. The enriched functions and pathways (*P* < 0.05) was further plotted by The Gene Set Enrichment Analysis (GSEA) through the clusterProfiler package. An integrated analysis of multi-omics data related to CRC obtained from The Cancer Genome Atlas (TCGA) and the Clinical Proteomic Tumor Analysis Consortium (CPTAC) was employed by the Linkomics database (www.linkedomics.org) to investigate the relationship between eIF4B Ser93 phosphorylation and EMT biomarkers. The PPI information of the studied proteins was analyzed by the STRING software (http://string-db.org/).

### Statistical analysis

The data are expressed as mean±SEM from at least three independent experiments. Data were analyzed by two-tailed unpaired t-test between two groups and One-way ANOVA among the multiple groups through GraphPad Prism software. Statistical significance was determined at *p < 0.05, **p < 0.01, ***p < 0.001, ****p < 0.0001.

## Supplementary information

The information of colorectal cancer tissue samples applied in this study

Supplementary material 2

Supplementary Figure legends

Figure S1

Figure S2

Figure S3

Figure S4

Figure S5

Figure S6

Figure S7

Uncropped images of blots

## Data Availability

The data generated and analyzed during the current study are available from the corresponding author on reasonable request.
