# Supplementary material for: EIF4B Ser93 phosphorylation by ERK2 promotes epithelial-mesenchymal transition to drive colorectal cancer metastasis
Source: Cell Death Dis. 2026 Jan 5;17(1):178. doi: 10.1038/s41419-025-08375-5 (PMC12877161; doi:10.1038/s41419-025-08375-5)
Supplement: Supplementary file 2 — Supplementary material 2 [file 41419_2025_8375_MOESM2_ESM.docx]

| Term | Category number | Brand |
| --- | --- | --- |
| eIF4B | 17917-1-AP | proteintech |
| N-cadherin | sc-53488 | Santa cruz |
| Snai1 | sc-271977 | Santa cruz |
| Vimentin | sc-66002 | Santa cruz |
| E-cadherin | 3195 | Cell Signaling Technology |
| Ki-67 | ab16667 | Abcam |
| Ubiquitin | 10201-2-AP | proteintech |
| β-actin | sc-58673 | Santa cruz |
| α-tublin | 3873 | Cell Signaling Technology |
| ERK2 | sc-1647 | Santa cruz |

**Supplementary Table 1: Antibodies used**

**Supplementary Table 2: Primer sequences**

| Gene | Species | Sequence |
| --- | --- | --- |
| eIF4B | Human | F-AGAAGTAAGTCAGACCAGGATGC |
|  |  | R- GAGGGGCTGGCATTACCTTT |
| N-cad | Human | F-AGCCAACCTTAACTGAGGAGT |
|  |  | R-GGCAAGTTGATTGGAGGGATG |
| Vim | Human | F- GCCCTAGACGAACTGGGTC |
|  |  | R- GGCTGCAACTGCCTAATGAG |
| Snai1 | Human | F-GGTTCTTCTGCGCTACTGCT |
|  |  | R-TGCTGGAAGGTAAACTCTGGAT |
|  |  | R- CAGCTTGCCATCTTGGAGTC |
| FN1 | Human | F- CCGCCGAATGTAGGACAAGA |
|  |  | R- TGTCAGAGTGGCACTGGTAG |

**Supplementary Table 3**

| Binding titer of non-phosphorylated peptides | |
| --- | --- |
| Dilution ratio | Purified antibody |
| 1K | 0.097 |
| 2K | 0.073 |
| 4K | 0.062 |
| 8K | 0.063 |
| 16K | 0.091 |
| 32K | 0.078 |
| 64K | 0.049 |
| 128K | 0.00126 |

**Supplementary Table 4**

| Binding titer of phosphorylated peptides | |
| --- | --- |
| Dilution ratio | Purified antibody |
| 1K | 2.44 |
| 2K | 1.961 |
| 4K | 1.384 |
| 8K | 0.899 |
| 16K | 0.6 |
| 32K | 0.468 |
| 64K | 0.406 |
| 128K | 0.448 |
