# Supplementary material for: EIF4B Ser93 phosphorylation by ERK2 promotes epithelial-mesenchymal transition to drive colorectal cancer metastasis
Source: Cell Death Dis. 2026 Jan 5;17(1):178. doi: 10.1038/s41419-025-08375-5 (PMC12877161; doi:10.1038/s41419-025-08375-5)
Supplement: Supplementary file 3 — Supplementary Figure legends [file 41419_2025_8375_MOESM3_ESM.docx]

**Supplementary Figure legends**

**Figure S1:** A-D. The proteins phosphorylation increasing group were significantly enriched on the cellular functions, such as regulation of cell-substrate adhesion, cell adhesion, substrate adhesion-dependent cell spreading and regulation of cell migration.

**Figure S2:** The mRNA level of eIF4B was measured by RT‒qPCR in shNC and sheIF4B cells.

**Figure S3:** A. The number of migrated cells and invaded cells of shNC and sheIF4B groups were presented as normalized values. B. The quantification of wound closure were presented. Error bars represent mean±SEM from at least three independent experiments. *P* values were determined by two-sided Student t test. **P*< 0.05, ***P*< 0.01, ****P*< 0.001, and *****P*< 0.0001.

**Figure S4:** A. The number of migrated cells and invaded cells in eIF4B WT, eIF4B S93A, and eIF4B S93D groups were presented as normalized values. B. The quantification of wound closure of these three groups. Error bars represent mean±SEM from at least three independent experiments. *P* values were determined by two-sided Student t test. **P*< 0.05, ***P*< 0.01, ****P*< 0.001, and *****P*< 0.0001.

**Figure S5:** Pearson correlation analysis of eIF4B Ser93 phosphorylation and EMT markers, such as CDH1 (E-cad), Twist1, MMP1, MMP3.

**Figure S6:** The quantification of eIF4B expression via western blotting were conducted following 0, 3, 6, and 12 hours of CHX treatment across different cell groups. At least three independent experiments were performed to quantify the eIF4B expression levels.

**Figure S7:** A. Schematic illustration of subcutaneous xenograft tumor model in nude mice. B. Schematic illustration of lung metastasis model via tail vein injection in nude mice.
