# Supplementary figures and images for: EIF4B Ser93 phosphorylation by ERK2 promotes epithelial-mesenchymal transition to drive colorectal cancer metastasis
Source: Cell Death Dis. 2026 Jan 5;17(1):178. doi: 10.1038/s41419-025-08375-5 (PMC12877161; doi:10.1038/s41419-025-08375-5)

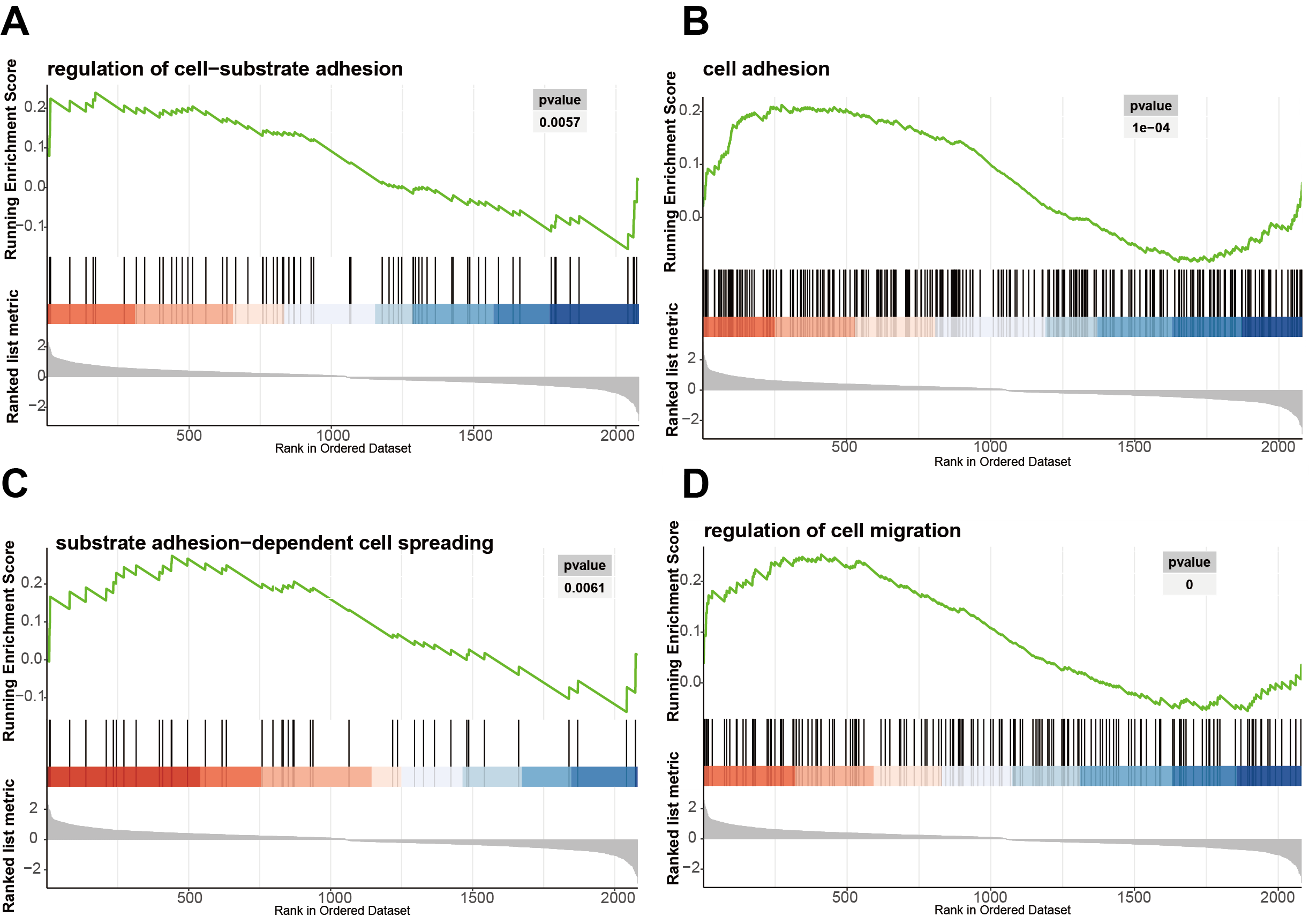

Supplement: Supplementary file 4 — Figure S1 [file 41419_2025_8375_MOESM4_ESM.tif]

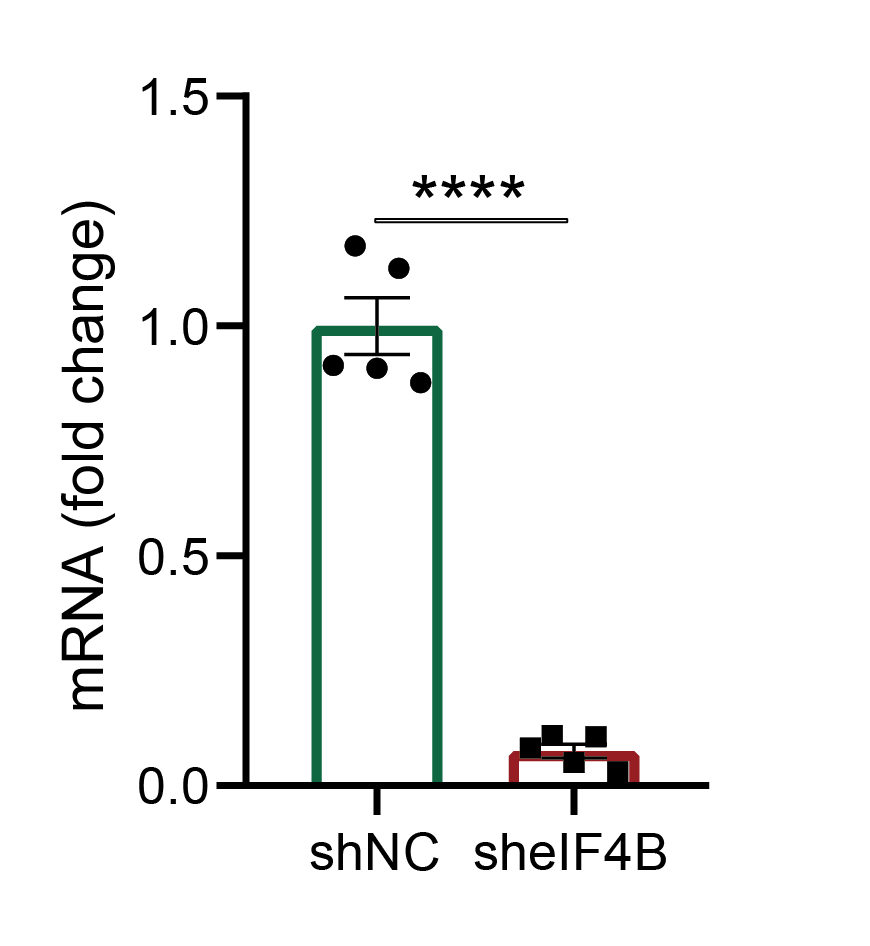

Supplement: Supplementary file 5 — Figure S2 [file 41419_2025_8375_MOESM5_ESM.tif]

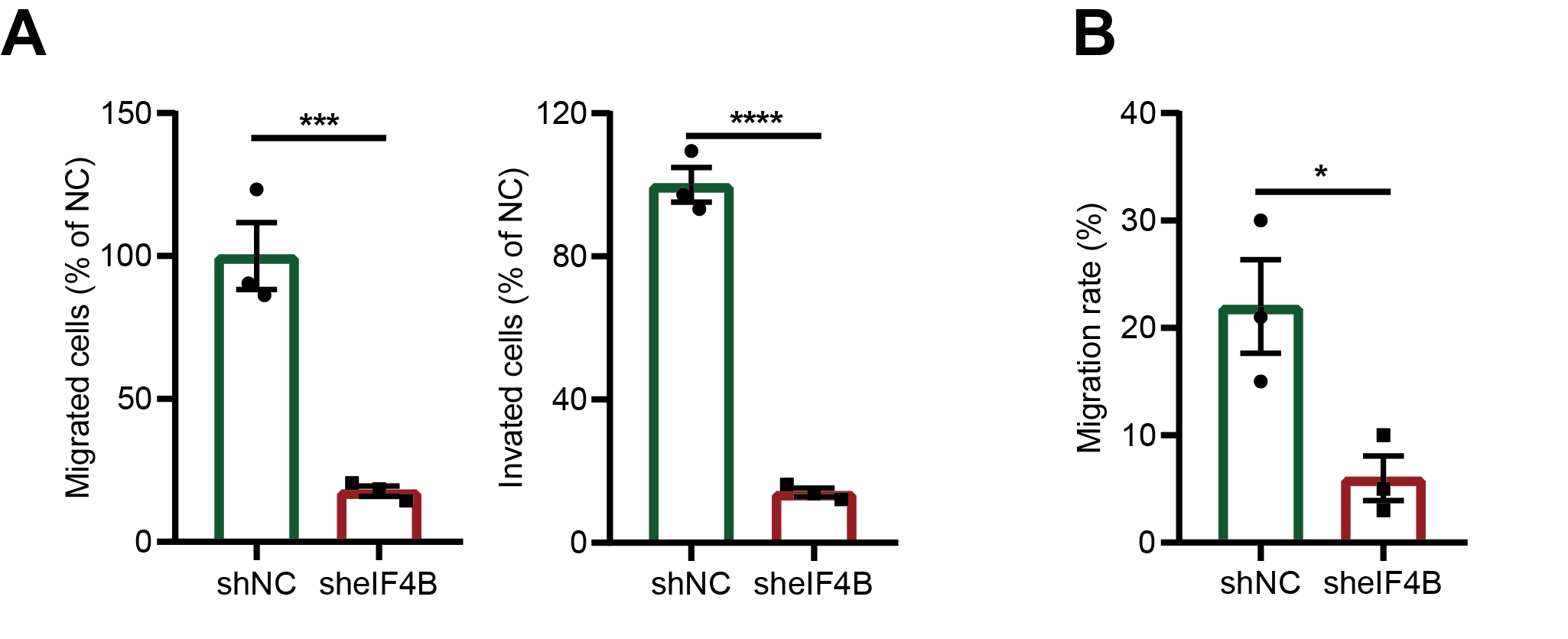

Supplement: Supplementary file 6 — Figure S3 [file 41419_2025_8375_MOESM6_ESM.tif]

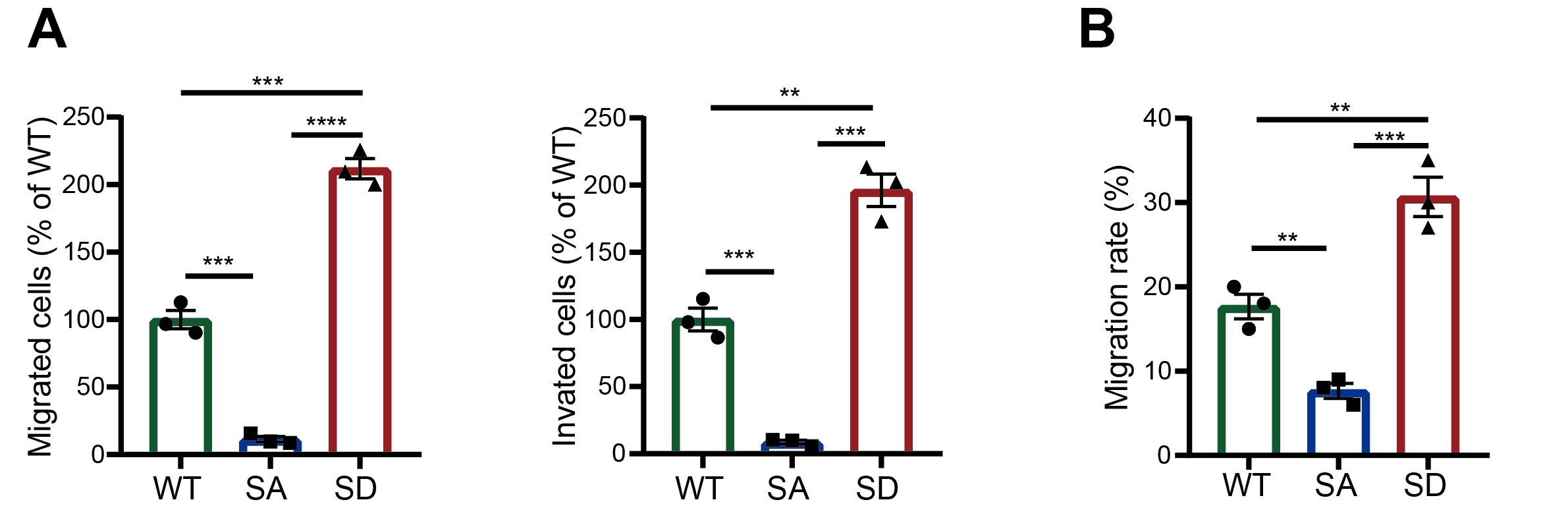

Supplement: Supplementary file 7 — Figure S4 [file 41419_2025_8375_MOESM7_ESM.tif]

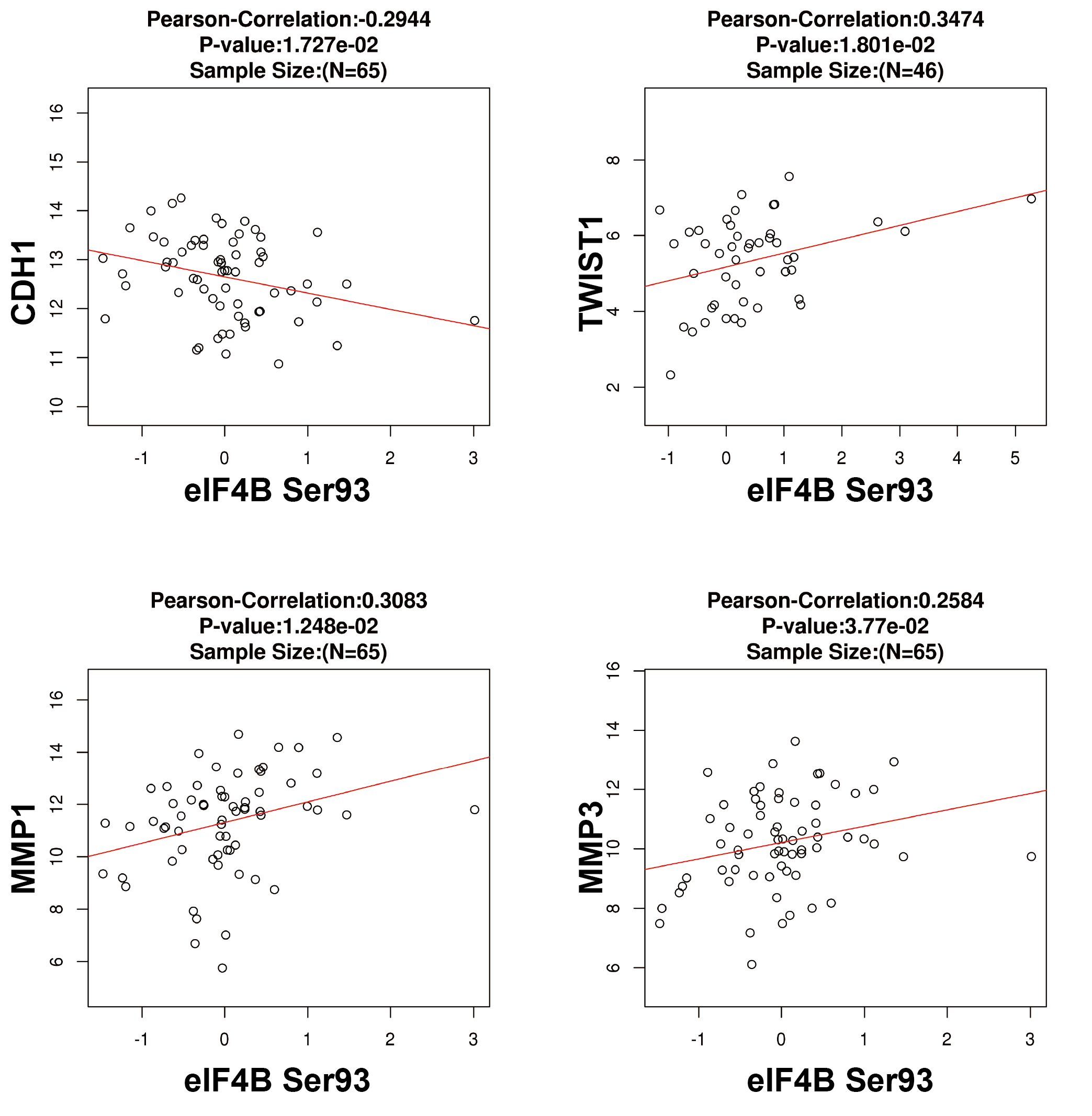

Supplement: Supplementary file 8 — Figure S5 [file 41419_2025_8375_MOESM8_ESM.tif]

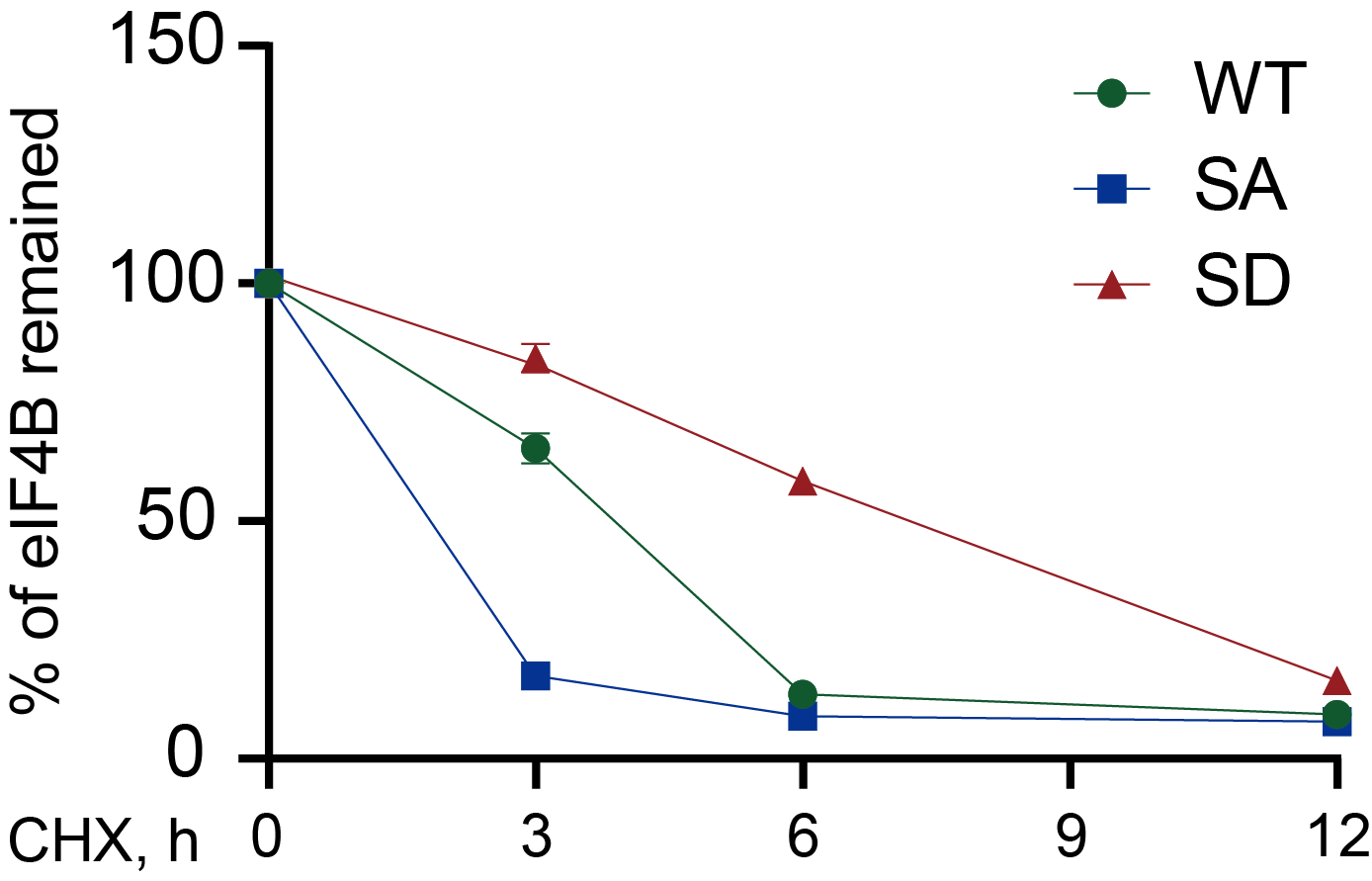

Supplement: Supplementary file 9 — Figure S6 [file 41419_2025_8375_MOESM9_ESM.tif]

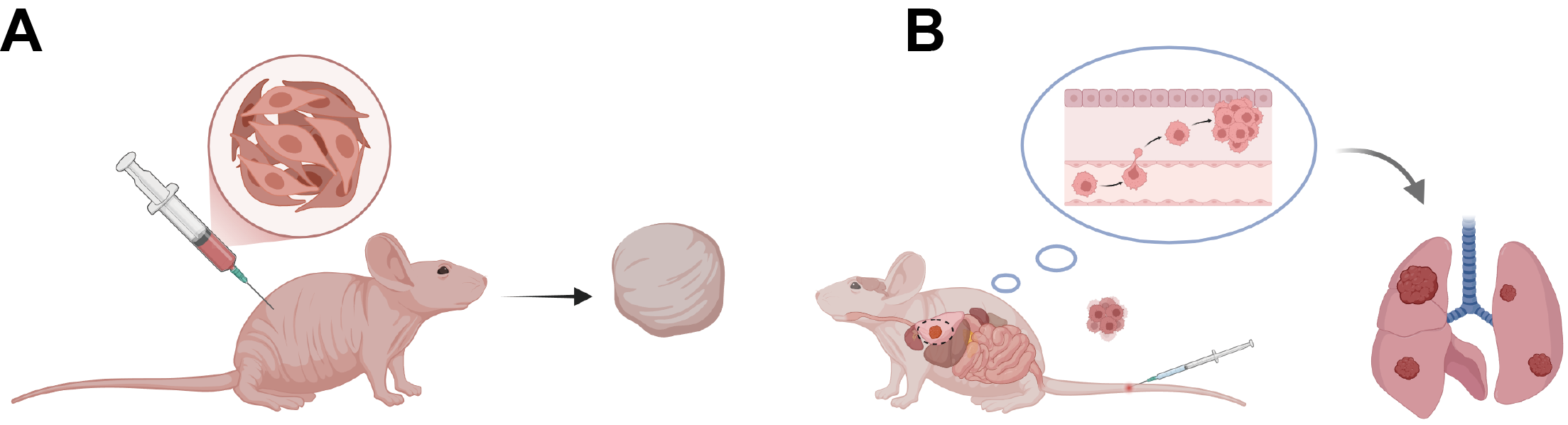

Supplement: Supplementary file 10 — Figure S7 [file 41419_2025_8375_MOESM10_ESM.tif]
