# Supplementary material for: EIF4B Ser93 phosphorylation by ERK2 promotes epithelial-mesenchymal transition to drive colorectal cancer metastasis
Source: Cell Death Dis. 2026 Jan 5;17(1):178. doi: 10.1038/s41419-025-08375-5 (PMC12877161; doi:10.1038/s41419-025-08375-5)
Supplement: Supplementary file 11 — Uncropped images of blots [file 41419_2025_8375_MOESM11_ESM.pptx]

## Slide 1
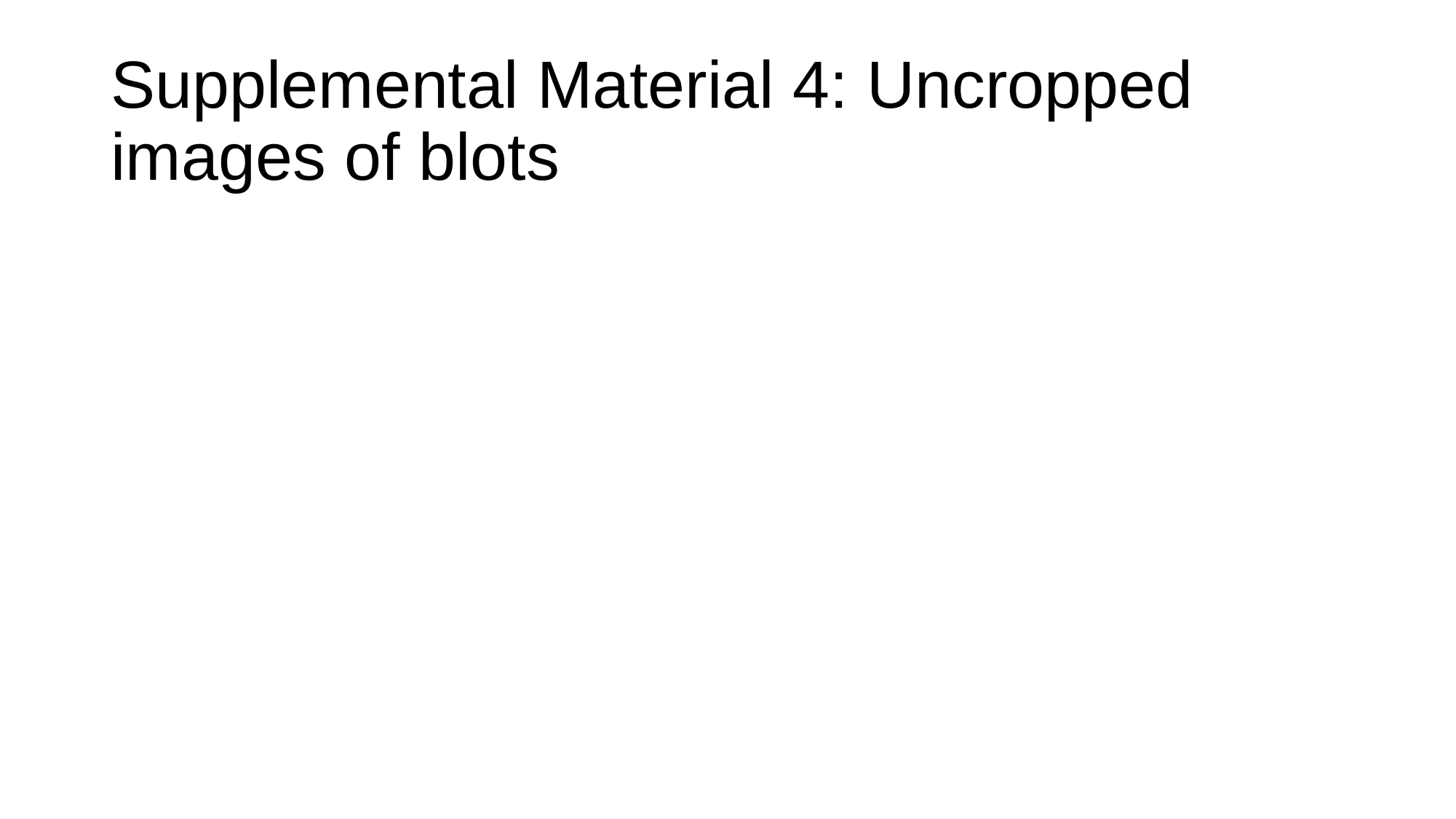

# Supplemental Material 4: Uncropped images of blots

## Slide 2
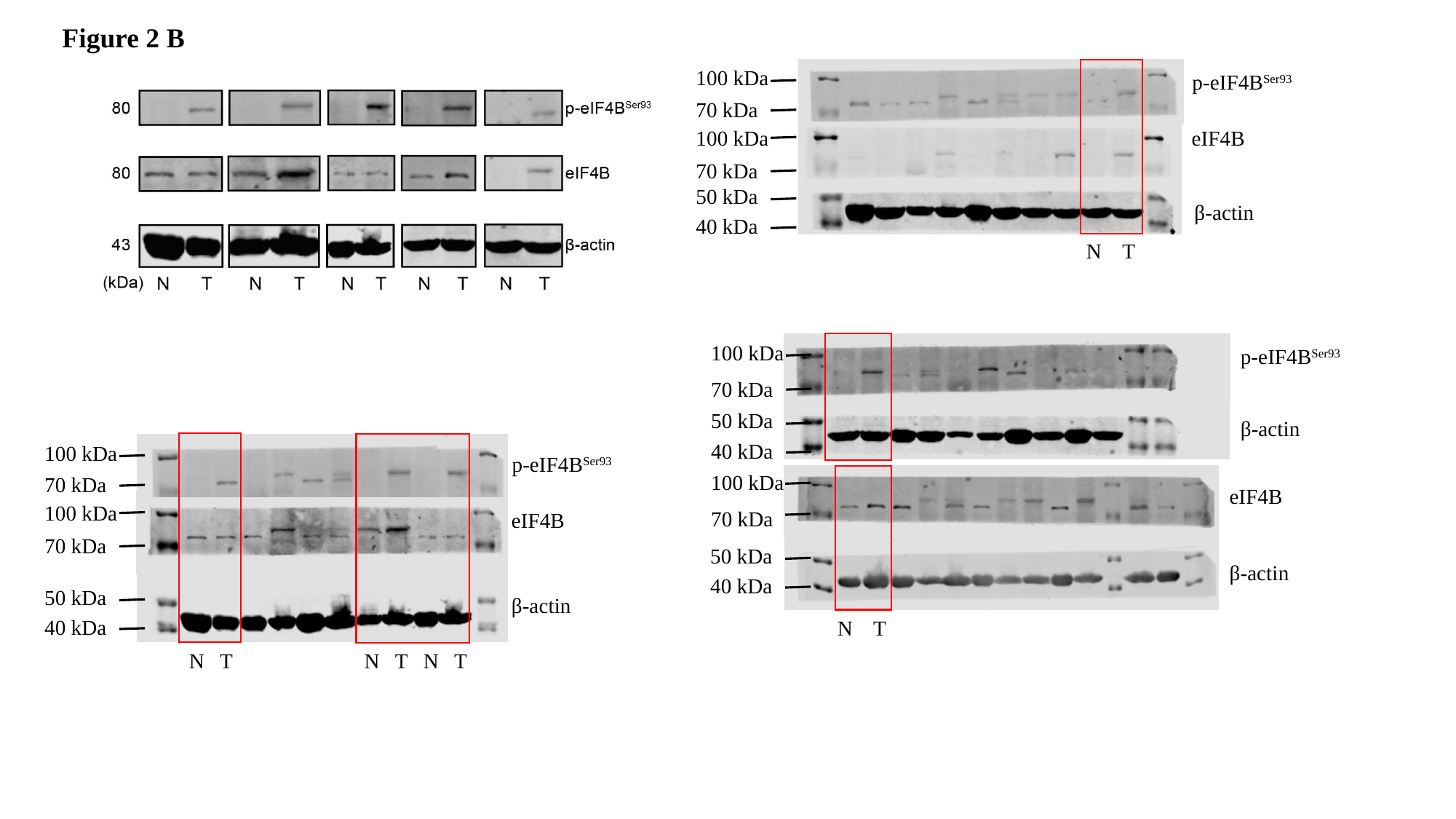

Figure 2 B
100 kDa
p-eIF4BSer93
70 kDa
eIF4B
100 kDa
70 kDa
50 kDa
β-actin
40 kDa
N T
100 kDa
p-eIF4BSer93
70 kDa
50 kDa
β-actin
40 kDa
100 kDa
p-eIF4BSer93
100 kDa
70 kDa
eIF4B
100 kDa
70 kDa
eIF4B
70 kDa
50 kDa
β-actin
40 kDa
50 kDa
β-actin
40 kDa
N T
N T N T N T

## Slide 3
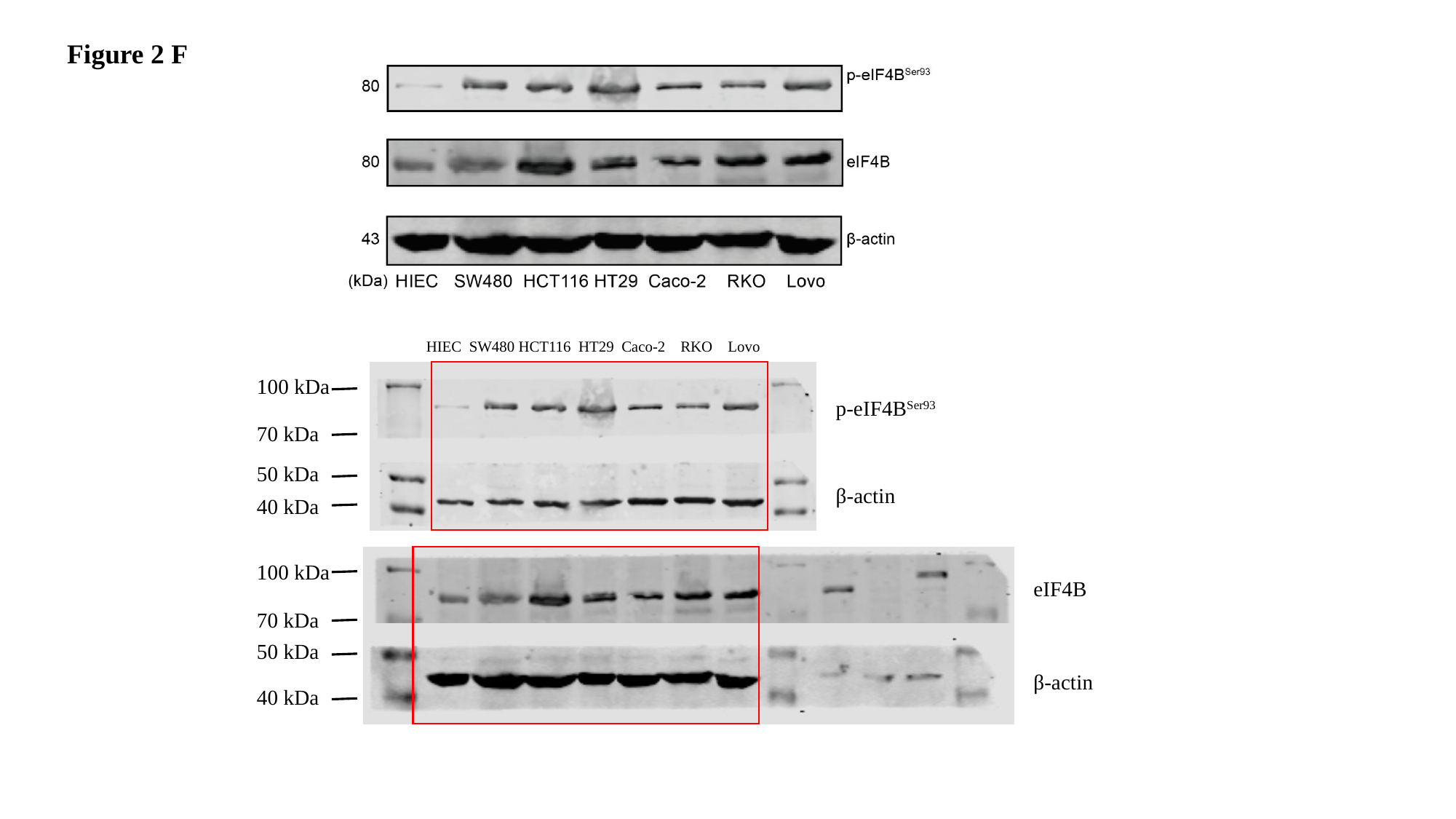

Figure 2 F
HIEC SW480 HCT116 HT29 Caco-2 RKO Lovo
100 kDa
p-eIF4BSer93
70 kDa
50 kDa
β-actin
40 kDa
100 kDa
eIF4B
70 kDa
50 kDa
β-actin
40 kDa

## Slide 4
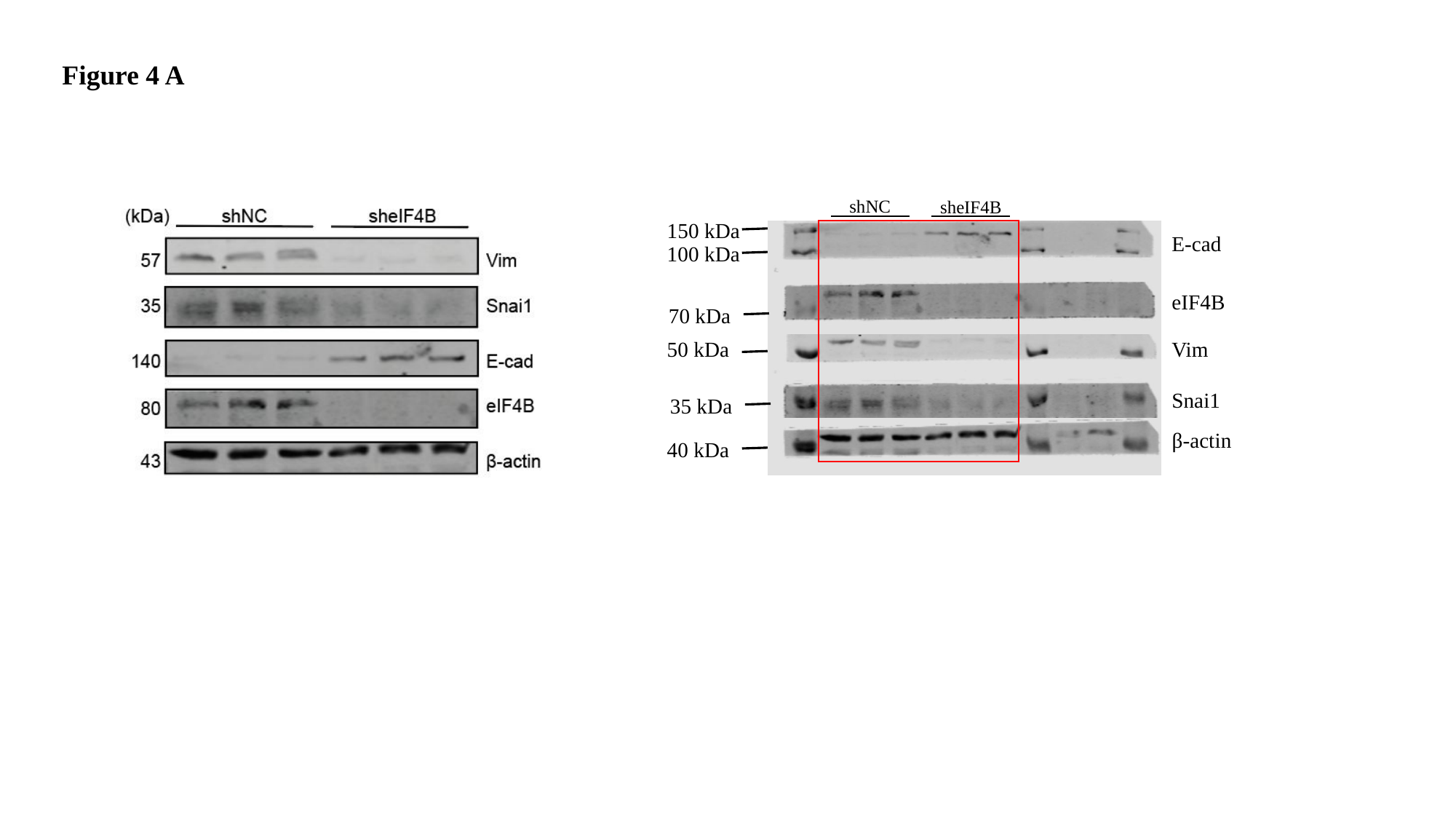

Figure 4 A
shNC
sheIF4B
150 kDa
E-cad
100 kDa
eIF4B
70 kDa
50 kDa
Vim
Snai1
35 kDa
β-actin
40 kDa

## Slide 5
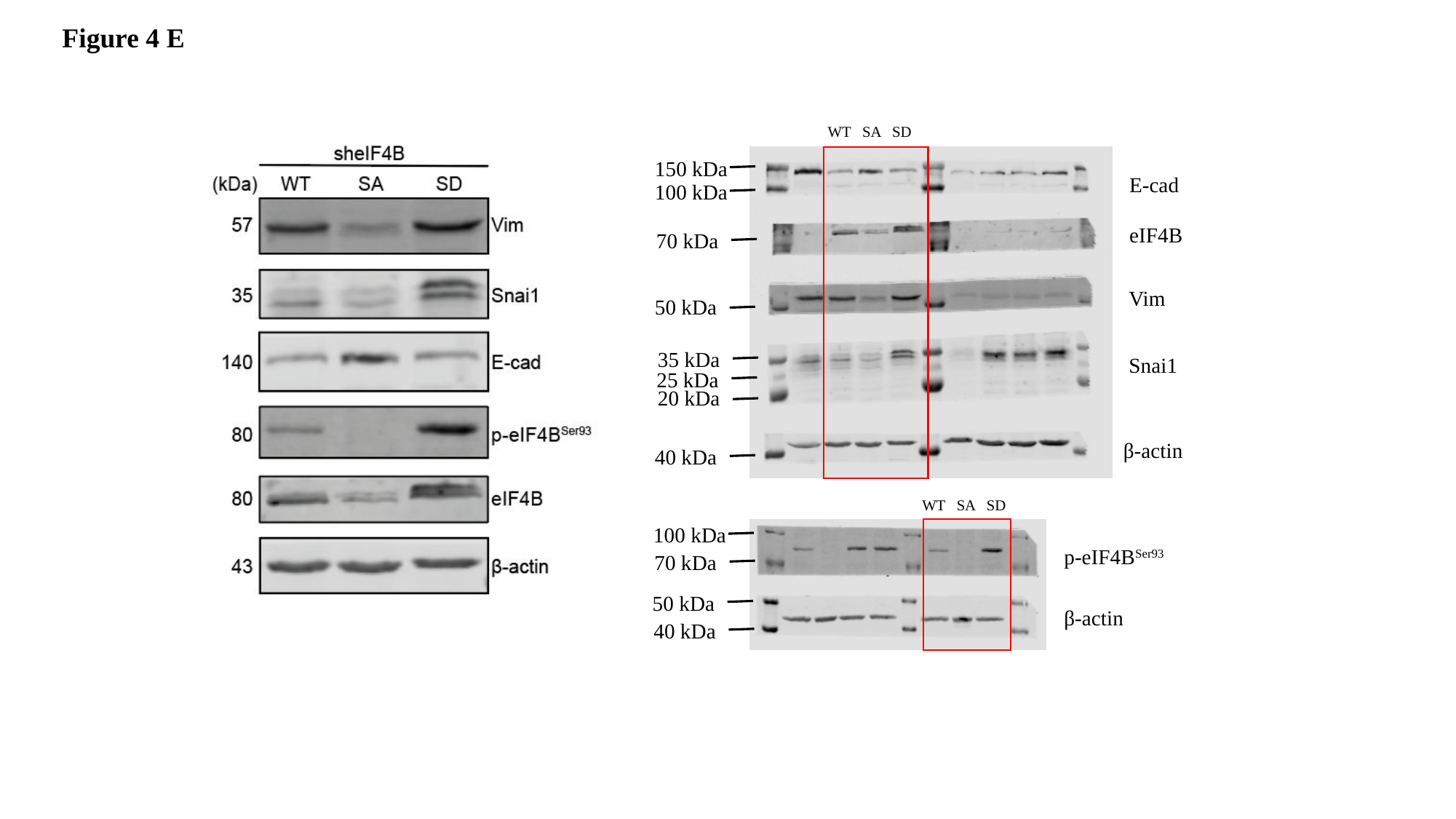

Figure 4 E
WT SA SD
150 kDa
E-cad
100 kDa
eIF4B
70 kDa
Vim
50 kDa
35 kDa
Snai1
25 kDa
20 kDa
β-actin
40 kDa
WT SA SD
100 kDa
p-eIF4BSer93
70 kDa
50 kDa
β-actin
40 kDa

## Slide 6
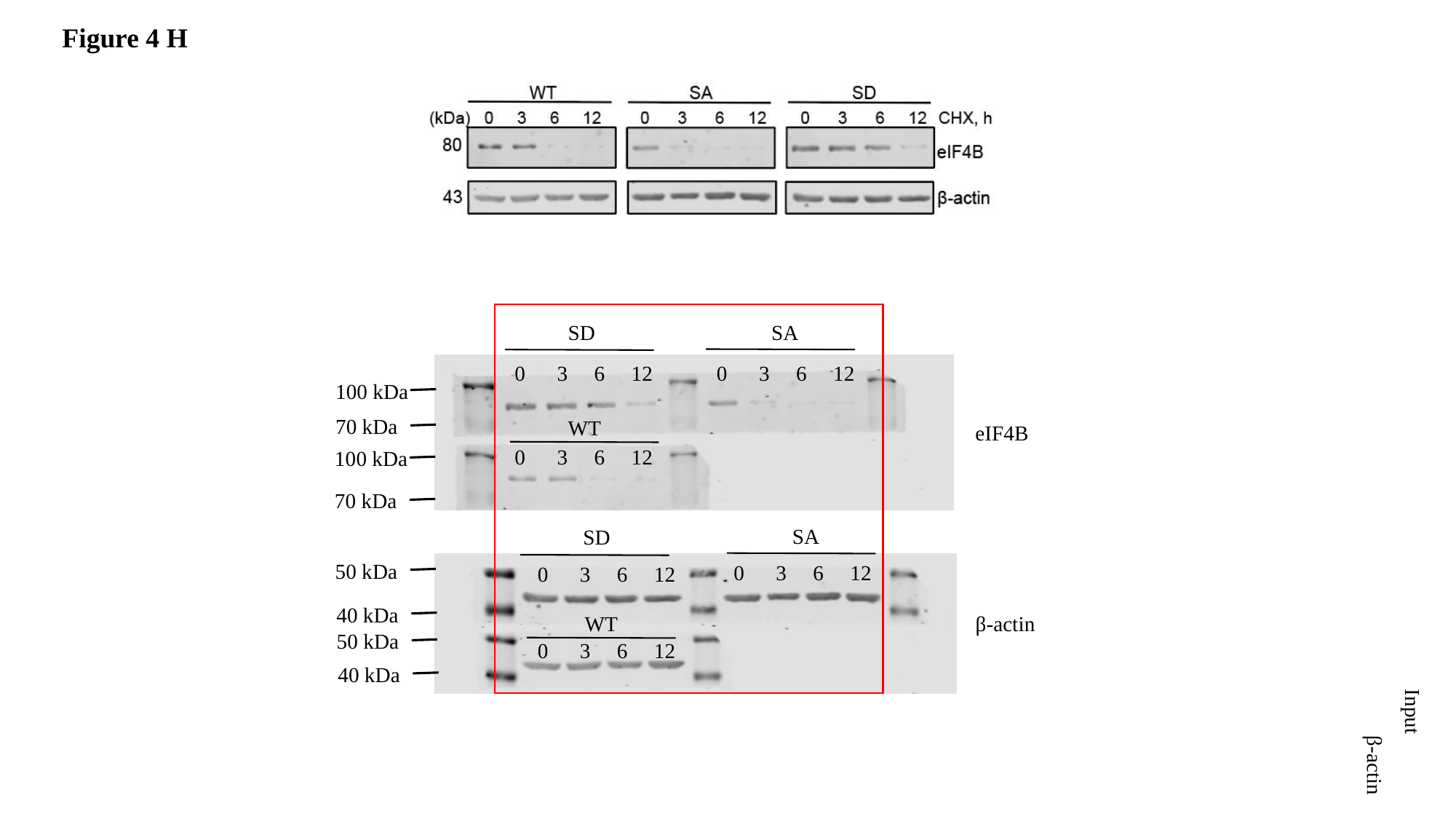

Figure 4 H
SD
SA
0 3 6 12
0 3 6 12
100 kDa
70 kDa
WT
eIF4B
0 3 6 12
100 kDa
70 kDa
SA
SD
50 kDa
0 3 6 12
0 3 6 12
40 kDa
WT
β-actin
50 kDa
0 3 6 12
40 kDa
Input
 β-actin

## Slide 7
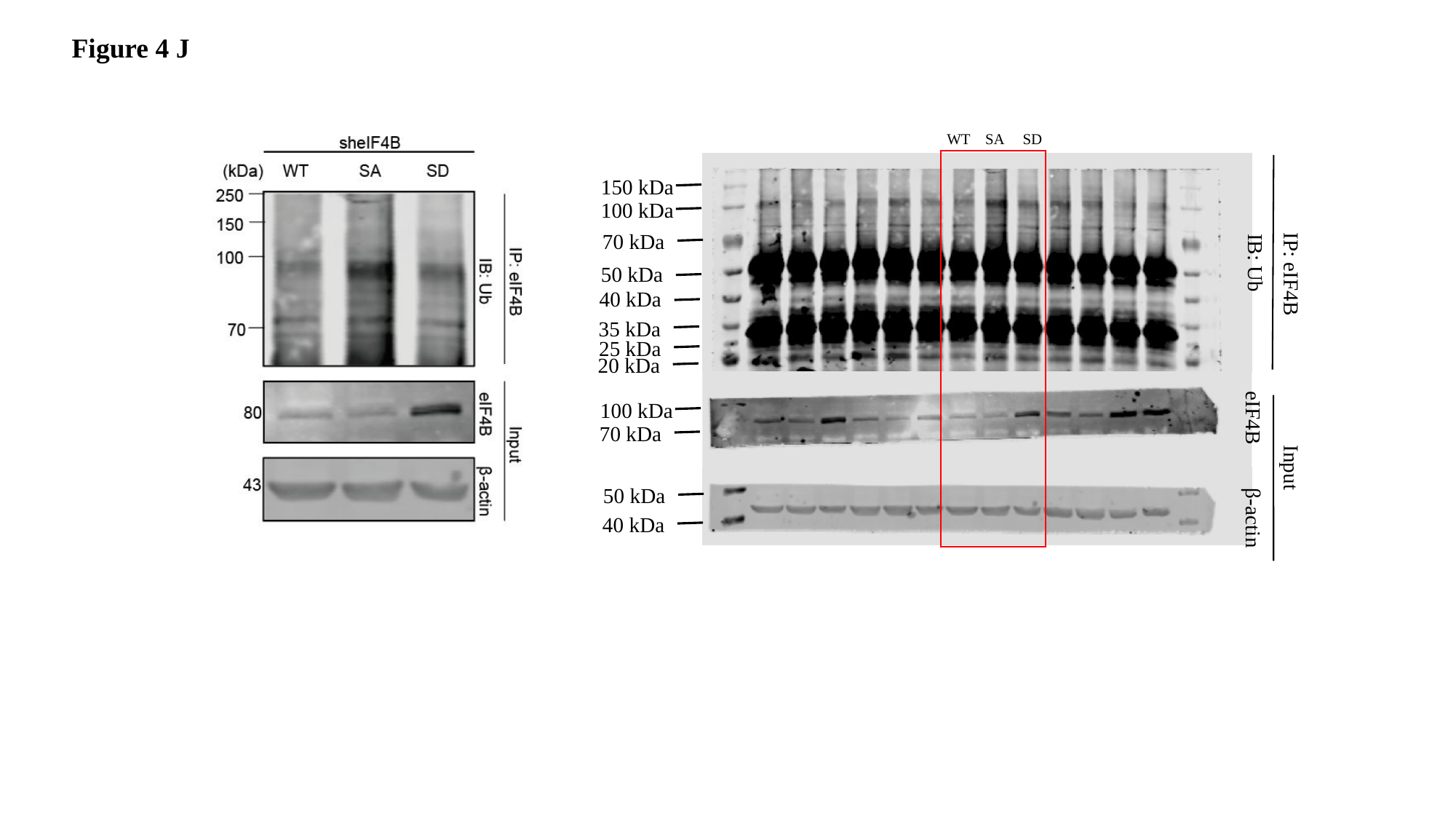

Figure 4 J
WT SA SD
150 kDa
100 kDa
70 kDa
IB: Ub
50 kDa
IP: eIF4B
40 kDa
35 kDa
25 kDa
20 kDa
100 kDa
 eIF4B
70 kDa
Input
50 kDa
 β-actin
40 kDa

## Slide 8
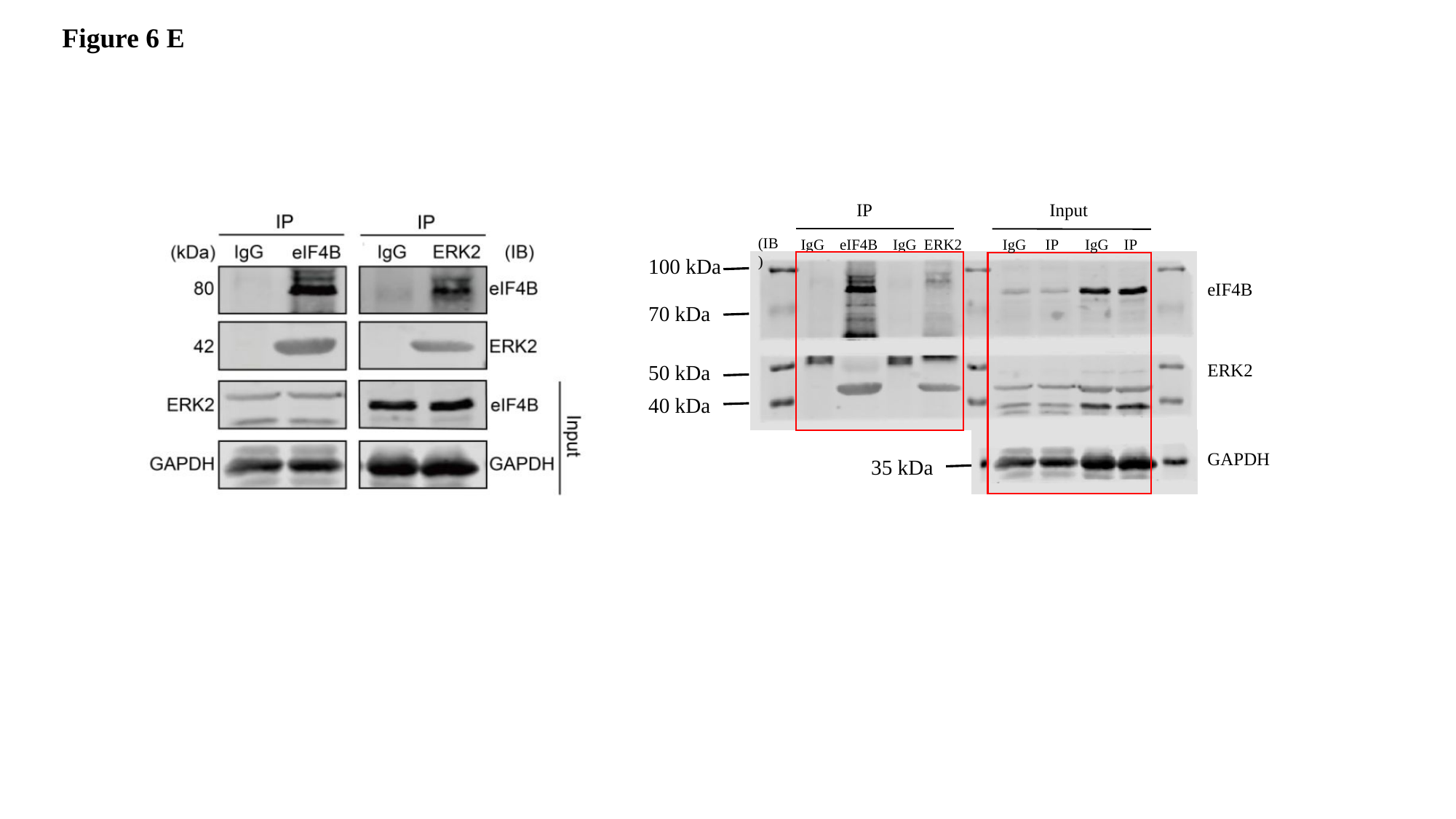

Figure 6 E
IP
Input
(IB)
IgG eIF4B IgG ERK2
IgG IP IgG IP
100 kDa
eIF4B
70 kDa
ERK2
50 kDa
40 kDa
GAPDH
35 kDa

## Slide 9
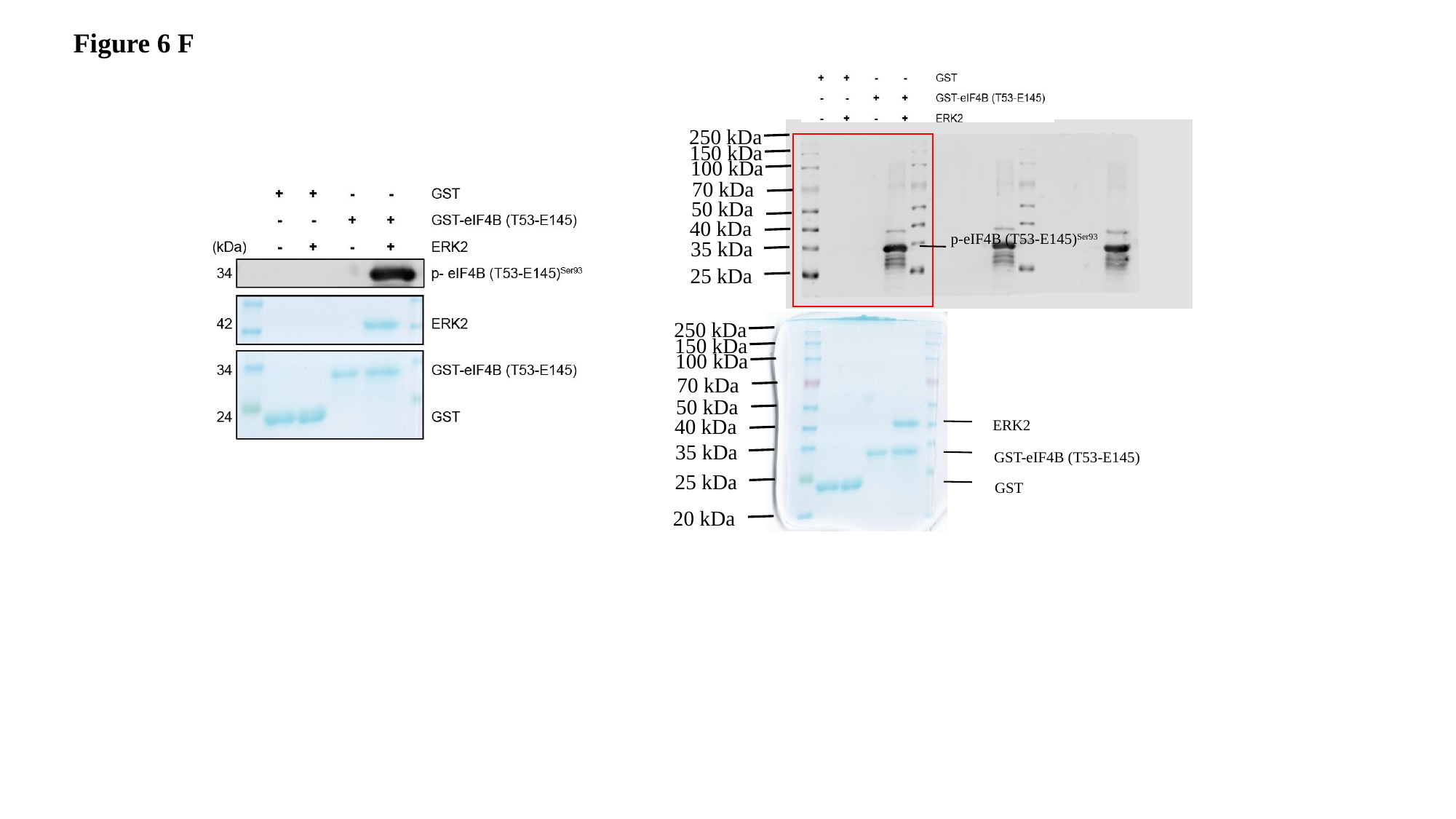

Figure 6 F
250 kDa
150 kDa
100 kDa
70 kDa
50 kDa
40 kDa
p-eIF4B (T53-E145)Ser93
35 kDa
25 kDa
250 kDa
150 kDa
100 kDa
70 kDa
50 kDa
40 kDa
ERK2
35 kDa
GST-eIF4B (T53-E145)
25 kDa
GST
20 kDa

## Slide 10
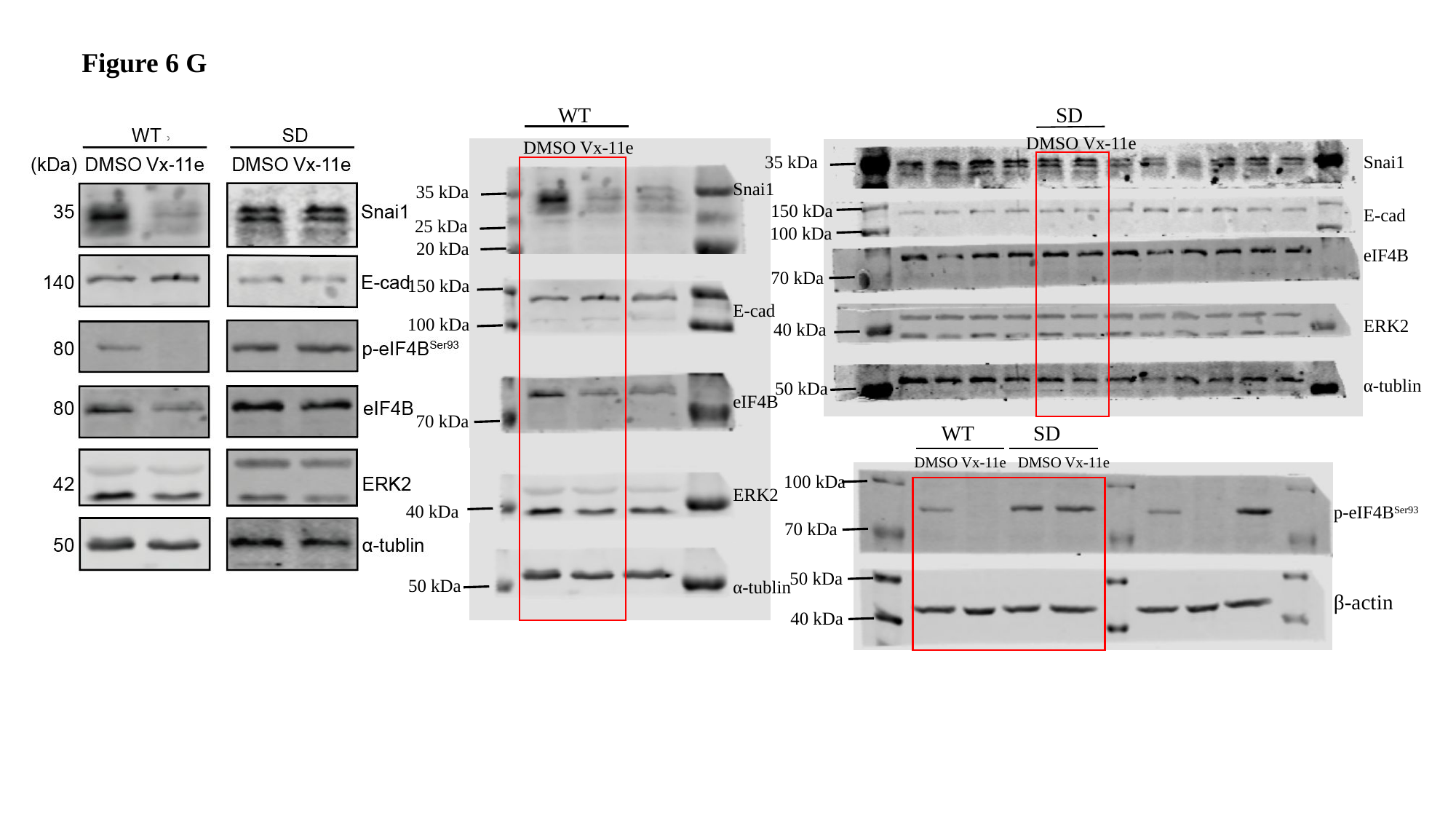

Figure 6 G
WT
SD
DMSO Vx-11e
DMSO Vx-11e
35 kDa
Snai1
Snai1
35 kDa
150 kDa
E-cad
25 kDa
100 kDa
20 kDa
eIF4B
70 kDa
150 kDa
E-cad
100 kDa
ERK2
40 kDa
α-tublin
50 kDa
eIF4B
70 kDa
WT
SD
DMSO Vx-11e
DMSO Vx-11e
100 kDa
ERK2
40 kDa
p-eIF4BSer93
70 kDa
50 kDa
50 kDa
α-tublin
β-actin
40 kDa
